# Post-COVID Complications after Pressure Ulcer Surgery in Patients with Spinal Cord Injury Associate with Creatine Kinase Upregulation in Adipose Tissue

**DOI:** 10.3390/cells11081282

**Published:** 2022-04-09

**Authors:** Mario Martínez-Torija, Pedro F. Esteban, Francisco Javier Espino-Rodríguez, Beatriz Paniagua-Torija, Eduardo Molina-Holgado, Silvia Ceruelo, Gemma Barroso-Garcia, Alba G. Arandilla, Luis F. Lopez-Almodovar, Angel Arevalo-Martin, Juan Antonio Moreno, Daniel Garcia-Ovejero, Mª Carmen Durán-Ruiz, Rafael Moreno-Luna

**Affiliations:** 1 Laboratory of Neuroinflammation, Hospital Nacional de Paraplejicos (SESCAM), 45071 Toledo, Spain; mariomartineztorija@gmail.com (M.M.-T.); pfesteban@sescam.jccm.es (P.F.E.); espinoja@gmail.com (F.J.E.-R.); pantorbe@gmail.com (B.P.-T.); eduardom@sescam.jccm.es (E.M.-H.); aarevalom@sescam.jccm.es (A.A.-M.); dgarciao@sescam.jccm.es (D.G.-O.); 2 Department of Nursing, Hospital Universitario de Toledo (SESCAM), 45071 Toledo, Spain; 3 Plastic and Reconstructive Surgery Service, Hospital Nacional de Paraplejicos (SESCAM), 45071 Toledo, Spain; 4 Department of Physical Rehabilitation, Hospital Nacional de Paraplejicos (SESCAM), 45071 Toledo, Spain; scabajo@gmail.com; 5 Proteomics Core Facility, Hospital Nacional de Paraplejicos (SESCAM), 45071 Toledo, Spain; gbarroso@sescam.jccm.es (G.B.-G.); agarandilla@sescam.jccm.es (A.G.A.); 6 Cardiac Surgery, Hospital Universitario de Toledo (SESCAM), 45071 Toledo, Spain; lopezalmodovar@yahoo.es; 7 Maimonides Biomedical Research Institute of Cordoba (IMIBIC), UGC Nephrology, Hospital Universitario Reina Sofia, 14004 Cordoba, Spain; jamoreno@fjd.es; 8 Cell Biology, Physiology and Immunology Department, Agrifood Campus of International Excellence (ceiA3), University of Cordoba, 14014 Cordoba, Spain; 9 Biomedicine, Biotechnology and Public Health Department, University of Cadiz, 11002 Cadiz, Spain; 10 Institute of Biomedical Research Cadiz (INiBICA), 11002 Cadiz, Spain

**Keywords:** COVID-19, SARS-CoV-2, SCI, PU, surgery, adipose tissue, proteomics, CKs, MGCs, autoantibodies

## Abstract

The risk of complications following surgical procedures is significantly increased in patients with SARS-CoV-2 infection. However, the mechanisms underlying these correlations are not fully known. Spinal cord injury (SCI) patients who underwent reconstructive surgery for pressure ulcers (PUs) before and during the COVID-19 pandemic were included in this study. The patient’s postoperative progression was registered, and the subcutaneous white adipose tissue (s-WAT) surrounding the ulcers was analyzed by proteomic and immunohistochemical assays to identify the molecular/cellular signatures of impaired recovery. Patients with SCI and a COVID-19-positive diagnosis showed worse recovery and severe postoperative complications, requiring reintervention. Several proteins were upregulated in the adipose tissue of these patients. Among them, CKMT2 and CKM stood out, and CKM increased for up to 60 days after the COVID-19 diagnosis. Moreover, CKMT2 and CKM were largely found in MGCs within the s-WAT of COVID patients. Some of these proteins presented post-translational modifications and were targeted by autoantibodies in the serum of COVID patients. Overall, our results indicate that CKMT2, CKM, and the presence of MGCs in the adipose tissue surrounding PUs in post-COVID patients could be predictive biomarkers of postsurgical complications. These results suggest that the inflammatory response in adipose tissue may underlie the defective repair seen after surgery.

## 1. Introduction

Pressure ulcers (PUs) are one of the most frequent complications of spinal cord injury (SCI) [1]. While conservative treatments such as pressure redistribution and improvement of nutritional status are adequate at initial stages I and II [2], PU with poor evolution can only be addressed by surgical intervention.

The COVID-19 pandemic has had a strong impact on surgical operations. Not only has the number of interventions significantly decreased [3,4], but the number of related complications, days of hospitalization, and mortality in positive patients have also increased in the immediate preoperative period or at the time of surgery [5,6] and in the early postoperative period [7]. Different factors are involved in this adverse outcome, such as prothrombotic effects, vasculopathy, ischemia, and tissue damage associated with the coronavirus infection [8]. Similarly, PU-related complications (healing time, infections, etc.) have also increased in COVID-19-positive patients [9]. However, existing evidence does not clarify whether such complications are due to the PU pathophysiology itself or to extrinsic factors, such as delays in interventions or the care received [10], infections associated with gastrointestinal problems [10], or the use of medical instrumentation [11]. In our own experience, patients with SCI undergoing PU reconstructive surgery showed serious complications and a worse recovery if they had SARS-CoV-2 infection within 4 weeks prior to the intervention. Such situations compromise surgical decisions and clinical planning, with no specific prognostic biomarkers currently available to estimate the expected recovery time or optimal timing for surgical reconstruction. 

The presence of angiotensin-converting enzyme 2 (ACE2), a SARS-CoV-2 receptor widely expressed in adipose tissue [12], has promoted increasing interest in this tissue due to its potential association with the viral infection [13]. Adipose tissue not only plays a role in glucose and lipid metabolism but also responds to metabolic stress by releasing endocrine factors that regulate diverse processes involved in tissue repair [14]. However, although SARS-CoV-2 has already been reported in adipose tissue [15], no signs of this virus have been found in PU exudate yet [16].

In the current study, we aimed to determine the particularities of the adipose tissue surrounding ulcers in patients with SCI who suffered from SARS-CoV-2 infection in the early postoperative period, as well as to identify features that may help to understand the underlying processes and prognostic biomarkers to anticipate complications.

## 2. Materials and Methods

### 2.1. Study Population

Patients who underwent PU reconstructive surgery before and during the COVID-19 pandemic, from January 2019 to July 2021, at the Paraplegic National Hospital (Toledo, Spain) were included in this study. The study was approved by the local institutional review board (Ethics and Clinical Investigation Committee of the Toledo Hospital Complex, code 207, approval date DATA 14 February 2018) and followed the principles of the Declaration of Helsinki. In addition, personal data protection was ensured, as required by Spanish Law (Organic Law on Data Protection 3/2018). All patients provided informed consent before inclusion in the study.

### 2.2. Study Design

Patients of any sex with SCI and PUs (grade III or IV) who required reconstructive surgery between 2019 and 2021 were selected by screening our medical records. The patient’s available data included sex, age, SCI type, ulcer characteristics, comorbidities, SARS-CoV-2 infection status (if recruitment was performed after the onset of the COVID-19 pandemic) (Table 1), clinical diagnosis, and postoperative course (morbidity and atypical reinterventions). From the initial number of patients recruited (n = 23), those for whom we did not have sufficient data or who did not agree to participate in this study were excluded (n = 6).

Groups were defined as follows:

Group A (n = 7), pre-pandemic (2019): patients who required PU reconstructive surgery between 1 January and 30 December 2019, defined as the non-COVID group.

Group B (n = 10), during the pandemic period: patients who required PU reconstructive surgery from 1 January 2020 to 30 July 2021. This group was stratified by SARS-CoV-2 infection status (nasopharyngeal RT-qPCR) between COVID-19-negative (n = 4) and COVID-19-positive (n = 3) patients, defined as post-COVID. Patients without PCR test results were excluded from the study (n = 3). The final number of patients in Group B was 7. 

### 2.3. Surgical Procedure

During the initial surgical debridement, the unhealthy skin edge of the ulcer was marked, and the ulcer was excised to a healthy bleeding level. Immediately after reconstruction, patients were treated according to a postoperative protocol based on the criteria followed at the Paraplegic National Hospital (Toledo, Spain), a national reference center for the treatment of SCI and PU, which includes their accommodation in therapeutic postural beds. Photographs of the PUs were taken 1–2 days before surgery and 15–25 days after surgery. COVID patients waited for surgery until confirmation of a negative SARS-CoV-2 RT-PCR nasopharyngeal swab result. All surgical interventions were performed by the same surgeon and his team.

### 2.4. Biopsy Procedure

Open biopsy specimens were collected from the ulcerated tissue area and from an adjacent sample consisting of subcutaneous white adipose tissue (s-WAT) peripheral to the ulcer. To ensure the collection of the purest s-WAT, tissue was collected and resected at least 2 cm away from the ulcerated area. Immediately after removal, each tissue sample was divided into two pieces: one was fixed in 10% formalin for histological examination, and the other was snap-frozen in liquid nitrogen for further mRNA and protein extraction.

### 2.5. Blood Sera Collection

Peripheral blood was collected in anticoagulant-free tubes by venipuncture of the medial cubital vein. Blood clots were formed by maintaining the tubes at room temperature (RT) for 30 min. Blood was centrifuged at 1500× *g* for 10 min at 4 °C, and serum was collected, aliquoted, and stored at −80 °C until use.

### 2.6. Protein Isolation

Frozen adipose tissue was homogenized in 0.5 mL of lysis buffer (1 % (*w*/*v*) SDC; 10 mM TCEP; 100 mM TRIS; 40 mM chloroacetamide; protease and phosphatase inhibitors pH: 8.5), overnight at 4 °C with rotation. The samples were centrifuged at 13,000× *g* for 15 min at 2–8 °C. The top layer (adipocytes) was then discarded. The resulting liquid layer was isolated and the remaining pellets were discarded.

### 2.7. RNA Isolation

Frozen adipose tissue (50–100 mg) was homogenized with a power homogenizer in 1 mL of QIAzol lysis reagent (Qiagen Sciences, Madrid, Spain), and total RNA was obtained by chloroform extraction and precipitation with isopropanol.

### 2.8. SARS-CoV-2 RT-PCR, Immunohistochemistry, and Immunological and Proteomic Analysis

A detailed description of all of these processes can be found in Supplemental Digital Content.

## 3. Results

### 3.1. Clinical Characteristics of the Patients

The mean age of patients was similar in both groups (55.0 ± 2.8 vs. 53.1 ± 5.0; *p* = 0.75), as was the degree of ulceration (PU IV in 6/7 patients, in both groups). From the total number of individuals, 11 out 14 were paraplegic (79%), while the rest suffered from tetraplegia. In addition, half of them (4/7 and 3/7 in Groups A and B, respectively) had cardiovascular problems. The complete patient clinical and demographic variables are shown in Table 1.

### 3.2. Clinical Complications in PU Postsurgical Recovery of SCI COVID Patients

**Figure 1:**
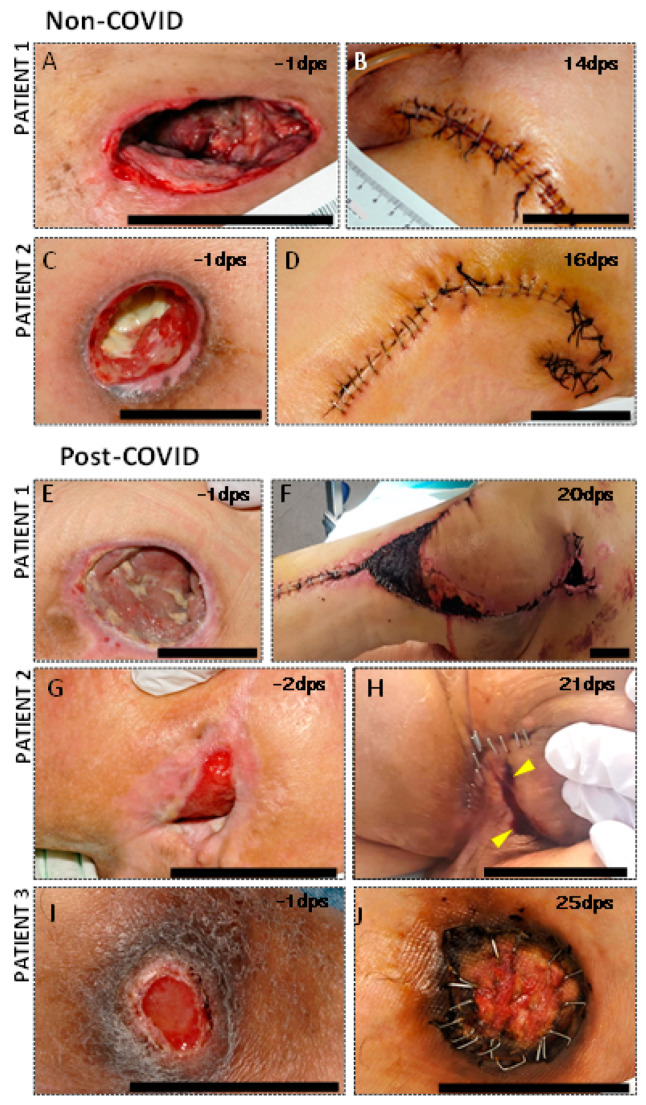
Pressure ulcers of non-COVID-19 and post-COVID-19 patients (**A**,**C**,**E**,**G**,**I**) and their evolution several days after undergoing surgery. DPS: days post-surgery. Note the worse appearance of all wounds in individuals who had suffered SARS-CoV-2 (**E**–**J**) compared to those who had not (**A**–**D**), including the presence of postsurgical hematomas (**F**), eschars, and necrotic areas (**F**,**H**,**J**), increased dehiscence risk (**H**,**J**), serum-hematic exudate through the surgical wound (**H**), and significantly delayed healing (**F**,**H**,**J**). The yellow triangles mark hematic and purulent exudate. Scale bar 5 cm.

SCI patients undergoing PU reconstructive surgery who were diagnosed as COVID-19-positive within 4 weeks prior to intervention showed more serious complications and a worse recovery than non-COVID-19 patients (Figure 1). In detail, patients who had suffered from SARS-CoV-2 approximately one month before surgical intervention presented a remarkable increase in serum hematic exudate through drains and the surgical wound, which lasted for more than three weeks. In addition, worse healing and wound dehiscence were apparent, despite removing the stitches after a month. Surgical reintervention was necessary for all patients. In one case, it was up to three times. Necrotic areas, eschars, and late postsurgical hematomas appeared approximately 20 days after the operation (Figure 1F). In contrast, no remarkable findings were detected in the recovery of patients who had not suffered from coronavirus infection, presenting uniform wound healing with a good approximation of tissue boundaries and no signs of infection. No alterations in the coagulation profile were observed in any of the patients.

### 3.3. The Subcutaneous White Adipose Tissue of Post-COVID Patients Does Not Show Presence of Viral Load

**Figure 2:**
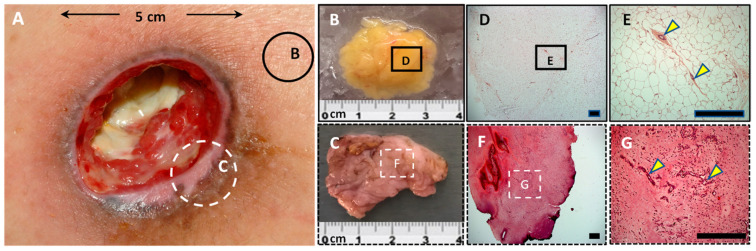
Pressure ulcers. (**A**) Representative image of pressure ulcers (PUs) in SCI patients. Macroscopic tissue: Biopsies were taken from the PU area, including a subcutaneous white adipose tissue (s-WAT) sample peripheral to the ulcer (**B**) and a sample from the ulcerated tissue area (**C**). Microscopic tissue: Histological sections analyzed in s-WAT (**D**,**E**) and ulcerated tissue (**F**,**G**) are shown. Blood vessels (yellow triangles) were also detected. Scale bars: 500 µm (**D**,**F**) and 100 µm (**E**,**G**).

S-WAT was easily differentiated from ulcerated tissue both macroscopically (Figure 2A,B,E) and histologically (Figure 2C,D,F,G) by the almost exclusive presence of adipocytes and sparse presence of vascular tissue (Figure 2C,D). Remarkably, no traces of SARS-CoV-2 were found in the s-WAT of COVID-19 patients: neither the expression of viral proteins (spike and nucleocapsid) by Western blotting nor viral load (RNA by RT-PCR) (data not shown) was detected. Thus, our results suggest that impaired wound healing is not associated with the presence of SARS-CoV-2 in the s-WAT.

### 3.4. Proteomic Analysis Revealed an Increased Presence of Creatine Kinase Isoforms in s-WAT of COVID Patients

**Figure 3:**
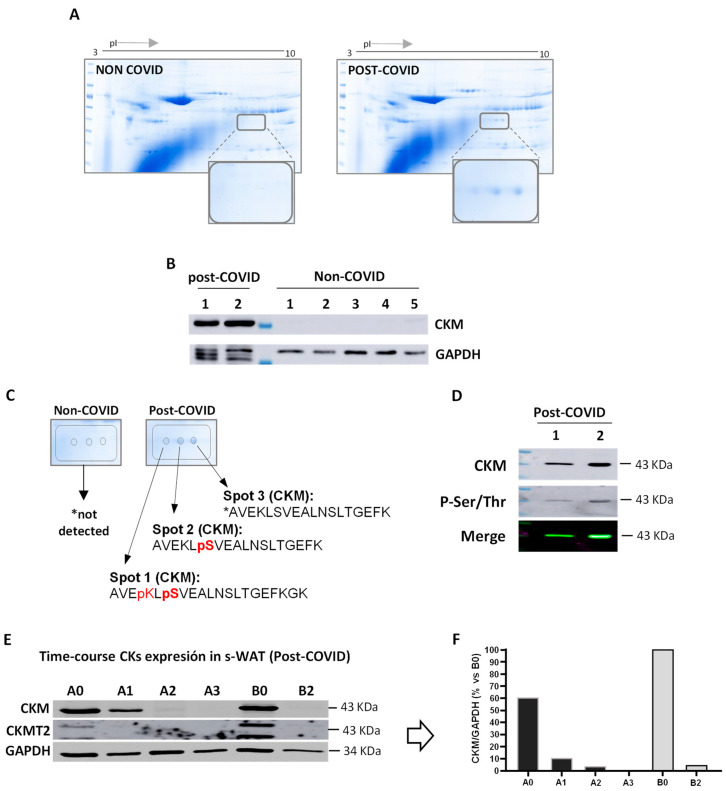
Proteomic analysis. (**A**) Representative 2D gels of s-WAT proteomes. The samples were analyzed in groups: s-WAT proteins from COVID-19-negative, pre-pandemic patients (non-COVID) and COVID-19-positive, post-pandemic patients (post-COVID). The inset shows the points (spots) extracted and analyzed. (**B**) Western blot of the MM isoforms of creatine kinase. GAPDH was used as load control. (**C**) Comprehensive analysis of the identified peptides shows the presence of post-translational modifications in lysine and serine residues. (**D**) Western blot confirming the presence of phosphorylation in serine residues. (**E**,**F**) Persistence of CKM and CKMT2 over time. (**E**) WB of CKM and CKMT2 from s-WAT of two post-COVID patients (A and B) at different periods of time. A0 and B0, first surgery; A1, surgical intervention approximately one month later; A2 and B2, surgical intervention approximately two months later. (**F**) Representative graph of the CKM and CKMT2 levels corrected by GAPDH and relativized to all highest values (B0).

Next, we aimed to explore the differential expression of proteins in the adipose tissue peripheral to the ulcers of these patients to gain insights into the underlying processes that lead to impaired wound healing. For this, we analyzed s-WAT proteins from COVID-19-negative (pre-pandemic, n = 7) and post-COVID-19 patients (n = 2) by 2D-WB (Figure 3). Our results showed the presence of three spots that were only detectable in samples from post-COVID patients (Figure 3A). Among the proteins identified by mass spectrometry (MS), creatine kinase (CK) isoforms CKM and CKMT2 were abundant in these spots (Figure 3A). To confirm this observation, we took an alternative approach: a control group of negative SARS-CoV-2 patients without SCI/PU was included (Supplementary Table S1), and the total s-WAT protein content from each group was analyzed by MS. The results obtained confirmed the previous data and also showed the presence of creatine kinase B-type isoform (CKMB) (Supplementary Figure S1). This isoform was present in all three groups; however, a greater number of peptides was identified in the post-COVID group (Supplementary Figure S1). We also detected the CKMT2 isoform exclusively in the post-COVID group, as well as a greater number of peptides for CKM than in the non-COVID group (Supplementary Figure S1).

A comprehensive analysis of the identified peptides showed the presence of post-translational modifications (PTMs) in lysine and serine residues (Figure 3C). The detection of phosphorylation sites in CKMT2 and CKM only in s-WAT from COVID patients was confirmed by colocalization in the Western blot (Figure 3D). CKMT2 colocalization was also observed with Ser/Thr (data not shown).

Finally, due to postsurgical healing problems and the need for reintervention, additional samples were extracted from these patients after the initial surgery. It was observed that CKM and CKMT2 expression was transitory, as both isoforms were not further expressed after the second month (Figure 3E).

### 3.5. The Expression of CKM and CKMT2 in COVID s-WAT Is Found in Multinucleated Giant Cells

**Figure 4:**
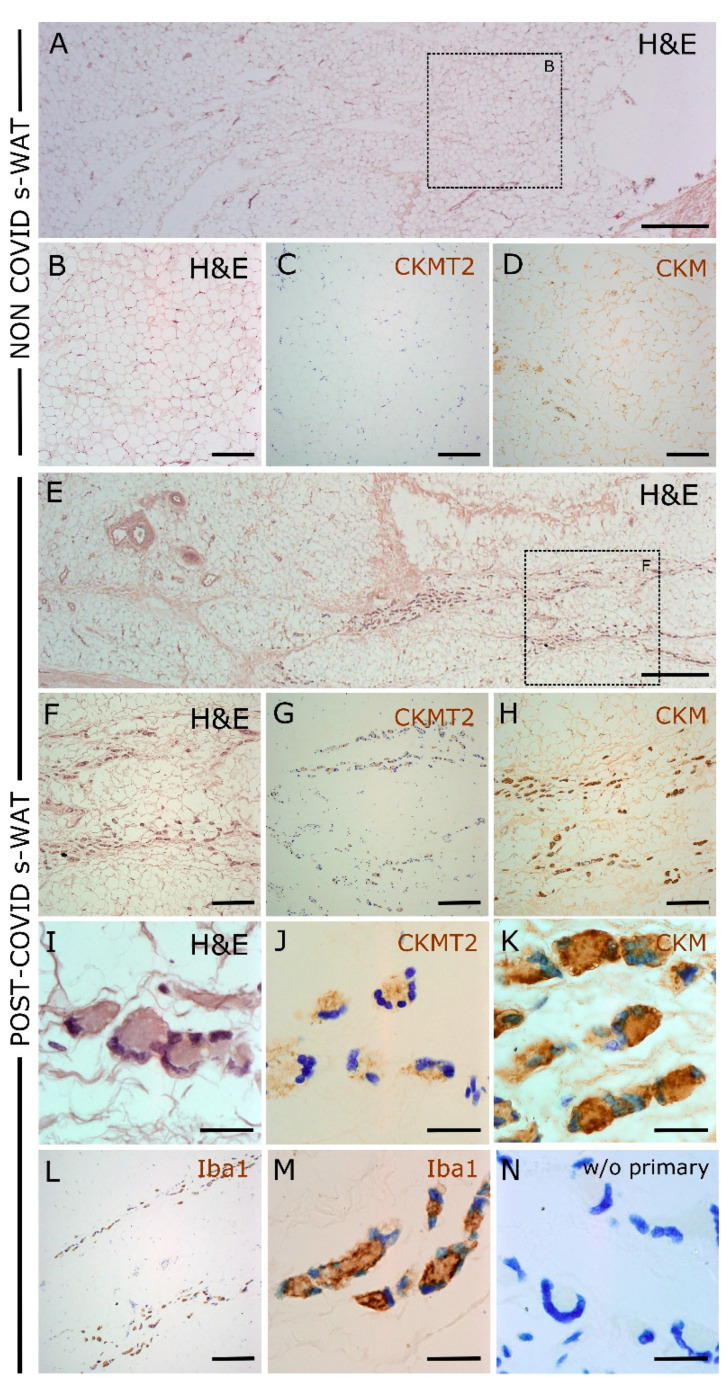
Immunohistochemical analysis of post-COVID and non-COVID patients’ s-WAT samples. (**A**) Low-magnification image of H&E staining, representative of non-COVID patients, showing normal-appearing adipose tissue. (**B**–**D**), higher-magnification details in which low or absent immunoreactivity of CKM (**C**) and CKMT2 (**D**) is observed. (**E**) Low-magnification image of H&E staining, representative of post-COVID patients, showing tissue distortion and accumulation of large macrophage-like cells (detail in (**F**)). A remarkable increase in CKM (**G**) and CKMT2 (**H**) immunoreactivity can be observed in post-COVID patients, as it is highly concentrated in these cells. (**I**–**K**) High-magnification images showing that CKM and CKMT2 immunoreactive cells are morphologically similar to multinucleated giant cells (MGCs). Mild toluidine blue counterstaining allowed the visualization of cell nuclei. (**L**,**M**) Cells expressing CKM and CKMT2 also express the macrophage marker Iba1. (**N**) The absence of primary antibody shows no specific staining. H&E, hematoxylin and eosin staining. Magnification bars: (**A**,**E**) 500 μm; (**B**–**D**,**F**–**H**,**L**) 100 μm; (**I**–**K**,**M**,**N**) 25 μm.

Immunohistochemistry assays confirmed the higher expression of CKM and CKMT2 in post-COVID patients (Figure 4). Although CKM expression was also found in adipocytes, the most notable feature was the presence of multinucleated giant cells (MGCs) in patients with COVID, with strong immunoreactivity against both CK isoforms (Figure 4J–K). The morphological aspect was consistent with a specific subtype of giant cells named Langhans-type giant cells: circular/ovoid-shaped, with a diameter below 50 microns and a limited number of nuclei, often arranged in a circular or semi-circular “horse-shoe” pattern [17]. These cells also expressed ionized calcium-binding adaptor molecule 1 (Iba1) (Figure 4), a protein that is specifically expressed by monocytic/macrophage lineages [18], confirming the macrophage nature of the CKM- and CKMT2-expressing cells.

### 3.6. Antibodies against Post-COVID-Induced Proteins Can Be Found in the Serum of Post-COVID Patients

**Figure 5:**
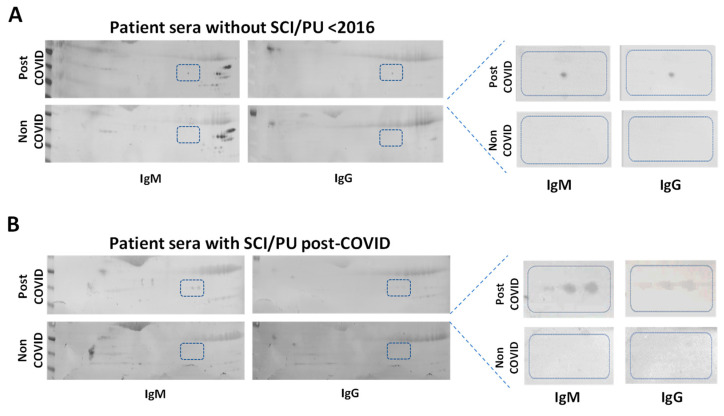
Evaluation of potential immunogenicity. (**A**,**B**) Representative 2D gels of s-WAT proteomes from both cohorts transferred to low-fluorescence PVDF membranes and incubated with sera from the different patient groups. The presence of autoantibodies is shown by the presence of dark spots using fluorescence-conjugated antibodies against human IgM and IgG. (**A**) The s-WAT of post-COVID (top) and non-COVID (bottom) patients, incubated with the serum of healthy volunteers without SCI and PU (all obtained before 2016). (**B**) The s-WAT of post-COVID (top) and non-COVID (bottom) patients, incubated with the serum of patients with SCI and PU post-COVID. Boxes with a dashed border indicate the marks selected in the study.

Finally, the potential immunogenicity of these post-translationally modified proteins was evaluated (Figure 5). For this, 2D gels from both groups were transferred to PVDF membranes and incubated with sera from non-COVID controls (obtained before 2016) or post-COVID patients (Figure 5). The presence of autoantibodies was determined by Western blotting.

After incubating the PVDF membranes containing proteins from the s-WAT of post-COVID patients with the serum of control subjects (without PU or SCI), serum IgGs and IgMs targeting the 2D spots at the intermediate isoelectric point were detected (Figure 5A). However, when these membranes (with the s-WAT proteins of post-COVID patients) were incubated with sera from post-COVID SCI patients suffering from PU, the three differentially expressed spots were targeted by autoantibodies (IgGs and IgMs) present in those sera (Figure 5B).

To elucidate whether these autoantibodies were caused by COVID infection, s-WAT proteins from COVID patients were exposed to sera from non-COVID patients with SCI and PU (also obtained before 2016, data not shown). Remarkably, the non-COVID sera showed immunoreactivity only against the same intermediate spot targeted by the control sera, suggesting that autoantibodies binding to the other spots were specifically detected only after COVID infection.

Neither of these signals was observed in membranes containing s-WAT proteins from non-COVID patients incubated with sera from non-COVID controls or post-COVID patients.

## 4. Discussion

The COVID pandemic has been a significant setback in the field of surgery. Beyond the large number of interventions that have been suspended and rescheduled [6], those that have been carried out present various associated complications, such as delayed healing, presence of purulent exudate, necrotic areas, and dehiscence of the edges, many requiring reintervention [5]. Recent studies have shown an increased risk of postoperative complications up to 4 weeks after SARS-CoV-2 infection, whereas surgery 8 weeks after COVID-19 was not associated with increased complications [7]. An increased risk of mortality has been observed too [19]. In this study, we compared the differential evolution of surgeries in SCI patients with PU in a post-COVID vs. non-COVID context. Notably, only the post-COVID patients with a positive PCR result for SARS-CoV-2 several weeks before surgery showed the complications previously described, while the rest recovered successfully. In addition, the recovery time for post-COVID patients was longer, and all required further intervention. An explanation for such complications could be a direct viral effect, since the coronavirus can enter adipocytes via ACE212; however, we did not find virus remnants, neither mRNA nor protein traces, in the s-WAT of these patients. For this, we planned an in-depth analysis based on multiple complementary techniques that could help us shed light on the reasons underlying this poor recovery.

Proteomic approaches revealed a significant increase in three CK isoforms in the s-WAT of post-COVID patients. CK is a ubiquitous enzyme with several isoforms [20]. Increased serum levels of total CK are a hallmark of severe muscle damage in critical patients [21], and circulating levels of the CKMB isoform (creatine kinase myocardial band) have been correlated with the severity of COVID-19 [22,23]. However, to our knowledge, no previous studies have reported on the local changes in CK isoforms in COVID-19 patients so far. We found the presence of CKMB in the s-WAT of all groups in this study, although it was more represented in post-COVID patients (Supplementary Figure S1). In addition, the CKM isoform was only found in SCI patients, with much higher levels in post-COVID patients, whereas the CKMT2 isoform was exclusively detected in post-COVID SCI patients. The overexpression of CKM and CKMT2 in the s-WAT of infected patients was transient, declining two months after COVID-19 diagnosis and correlating with the final recovery after surgical interventions. Likewise, transient elevation of serum CK levels has been described in mild and severe COVID-19 patients who showed high levels of CK at the time of hospitalization, returning to basal levels after several weeks [23]. Altogether, our data suggest that the presence of CKMT2 or CKM in the s-WAT of SCI/PU post-COVID patients within six weeks after infection may represent a prognostic biomarker of poor postsurgical recovery. In that case, delaying the intervention for several more weeks (6–8 weeks) until CKM or CKMT2 is no longer detected might be advised since this may increase the chances of a successful recovery.

Furthermore, CKM presented several PTMs, such as phosphorylation of Lys-156 and Ser-158, which were only found in patients with SCI and COVID-19. Previous studies have shown that CK phosphorylation regulates its activity and affects cellular concentrations of ATP, thereby affecting energy flux within cells [23,24,25]. Future studies are needed to validate this phenomenon in the context of SCI and post-COVID-19 complications.

Histological analysis of s-WAT from COVID patients revealed the presence of MGCs. MGCs are polykaryons of monocytic origin that arise in pathological lesions of widely varying etiology [26]. MGCs have long been regarded as hallmark indicators of chronic inflammatory processes, related either to a definable pathological agent (bacterial, viral, parasitic, or fungal infections) or to other cases when the inflammatory cause is not precisely known (rheumatoid arthritis, neoplasia) [17]. Relatively little is known about these cells [17,26]: they seem to be related to the persistent (unresolvable by normal phagocytosis) presence of foreign microorganisms or material, but their physiological significance is still unclear [17,26]. In adipose tissue, other macrophage formations, such as “crown-shaped structures”, are usually seen in inflammation [27,28], obesity [28,29], diabetes [27,30], and vascular damage [30]. The role of MGCs has been associated with the degradation of adipose tissue, especially dead adipocytes, but the existing literature does not clarify whether their presence is indicative of a good or poor prognosis [31]. Autopsies of COVID patients have reported the presence of macrophage infiltrates and MGCs in the lung [32,33,34], liver [35,36], kidney, spleen [34], and heart [36], but this is the first time, to our knowledge, that MGCs related to COVID infection have been found in adipose tissue. Since MGCs were only detected in post-COVID patients, these cells may also be associated with worse or prolonged recovery. As reported above, CKM and CKMT2 isoforms were highly expressed in the MGCs of COVID patients, which might reflect the metabolic state of these cells and may be involved in their activation, as described for macrophages [37,38]. In any case, together with the upregulation of CKM and CKMT2 isoforms, the presence of MGCs in s-WAT could be considered a potential adverse prognostic biomarker in post-COVID patients. Therefore, it may be used to establish a surgical strategy and schedule for a successful repair.

Finally, previous studies have reported a correlation between the presence of autoantibodies and a worse prognosis of the disease in COVID patients [39,40]. In our study, we observed the presence of autoantibodies against s-WAT proteins in the serum of patients with and without COVID. Some of these autoantibodies specifically recognized the spot detected at the same molecular weight where CK was identified. Among these autoantibodies, those directed against the most basic CK isoform were induced after COVID infection. The potential causes of these elevations are unclear. However, it has also been reported that the virus infects muscle cells (including cardiomyocytes) [41]. Myocytes express high levels of CK, and when myocytes are damaged, their contents are released (CK serum levels are commonly used as a marker of muscular damage). Thus, the increased levels of extracellular CK and/or the presence of PTMs in CK under cellular stress conditions may lead to the induction of an autoimmune response. Whether these autoantibodies are pathogenic or part of a healing response is a question that remains unanswered and deserves further study.

## 5. Conclusions

Overall, our results confirmed that patients with SCI who tested COVID-19-positive prior to surgery had worse recovery and severe postoperative complications, requiring reintervention. Moreover, the presence of MGCs, together with the overexpression of different CK isoforms found in the s-WAT of post-COVID patients, may be associated with the impaired wound healing observed. Given the limited number of samples that could be obtained for this study, future studies are required to confirm the results presented here. Nevertheless, our results suggest that both MGCs and high levels of CK isoforms might be considered potential signatures of adverse prognosis in SCI/PU patients infected with coronavirus.

## Figures and Tables

**Table 1:** Patient information registered included gender, age, SCI type, ulcer characteristics, and comorbidities. Abbreviations: SCI: spinal cord injury; PU: pressure ulcers; M: man; W: woman; P: paraplegic; T: tetraplegic; C: complete; I: incomplete; COVID: coronavirus disease; OSAHS: obstructive sleep apnea hypopnea syndrome.

	Patients	Age	Sex	SCI	Grade PU	PU Localization	Comorbidity
Pre-pandemic 2019	1	62	M	P/C	IV	Ischium	Hypomagnesemia, diabetic peripheral vascular disease dyslipidemia
	2	56	M	P/C	IV	Sacrum	Diabetic dyslipidemia
	3	43	M	P/I	IV	Heel	Arterial hypertension heart disease
	4	48	W	T/C	IV	Ischium	Respiratory insufficiency
	5	54	M	P/C	IV	Ischium	Arterial hypertension
	6	58	M	T/C	III	Trochanter	Respiratory insufficiency
	7	64	M	T/I	IV	Trochanter	Scoliosis pectus excavatum Respiratory insufficiency
Pandemic 2020–2021	1	67	M	P/C	IV	Trochanter	Diabetic obesity heart disease post-COVID-19
	2	53	M	P/C	IV	Ischium	Post-COVID-19
	3	39	M	P/C	III	Heel	Post-COVID-19
	4	71	W	P/C	IV	Ischium	Hypercholesterolemia
	5	50	M	P/C	IV	Trochanter	Osteoporosis
	6	35	M	P/C	IV	Ischium	Baclofen pump
	7	67	M	P/C	IV	Trochanter	Deep vein thrombosis OSAHS

## Data Availability

All relevant data are within the paper, together with online supplemental data files. In addition, proteomic results were deposited into the ProteomeXchange Consortium via the PRIDE partner repository (PXD031241). Deidentified data or other prespecified data will be available subject to a written proposal and signed data-sharing agreement.

## Supplementary Materials

The following supporting information can be downloaded at: https://www.mdpi.com/article/10.3390/cells11081282/s1, Supplementary Figure S1: Analysis of the total protein content of each group by LC-MS/MS; Supplementary Table S1: Clinical characteristics of the patients recruited in the control group, without SCI and PU; Supplementary Table S2: List of proteins identified in the three spots selected from the 2D gels of adipose tissue from non-COVID and post-COVID patients; Supplementary Table S3: List of peptides from creatine kinase M (CKM) and creatine kinase-S (CKS) isoforms identified in the three spots selected from the 2D gels of adipose tissue from non-COVID and post-COVID patients, as well as the post-translational modifications found in some of these peptides. Refs. 42,43,44,45 are cited in Supplementary Materials.

## References

[1] Zakrasek E.C., Creasey G., Crew J.D. (2015). Pressure ulcers in people with spinal cord injury in developing nations. Spinal Cord.

[2] Atkinson R.A., Cullum N.A. (2018). Interventions for pressure ulcers: A summary of evidence for prevention and treatment. Spinal Cord.

[3] Uimonen M., Kuitunen I., Paloneva J., Launonen A.P., Ponkilainen V., Mattila V.M. (2021). The impact of the COVID-19 pandemic on waiting times for elective surgery patients: A multicenter study. PLoS ONE.

[4] (2021). The Lancet Rheumatology Too long to wait: The impact of COVID-19 on elective surgery. Lancet Rheumatol..

[5] Bhangu A., Nepogodiev D., Glasbey J.C., Li E., Omar O.M., Gujjuri R.R., Morton D.G., Tsoulfas G., Keller D.S., Smart N.J. (2020). Mortality and pulmonary complications in patients undergoing surgery with perioperative sars-cov-2 infection: An international cohort study. Lancet.

[6] Collaborative C., Collaborative G. (2021). Timing of surgery following SARS-CoV-2 infection: An international prospective cohort study. Anaesthesia.

[7] Deng J.Z., Chan J.S., Potter A.L., Chen Y.-W., Sandhu H.S., Panda N., Chang D.C., Yang C.-F.J. (2022). The Risk of Postoperative Complications After Major Elective Surgery in Active or Resolved COVID-19 in the United States. Ann. Surg..

[8] Zhou F., Yu T., Du R., Fan G., Liu Y., Liu Z., Xiang J., Wang Y., Song B., Gu X. (2020). Clinical course and risk factors for mortality of adult inpatients with COVID-19 in Wuhan, China: A retrospective cohort study. Lancet.

[9] Black J., Cuddigan J., Capasso V., Cox J., Delmore B.M.N. (2020). Unavoidable Pressure Injury during COVID-19 Pandemic: A Position Paper from the National Pressure Injury Advisory Panel Previously the National Pressure Ulcer Advisory Panel (NPUAP) 1 ©NPIAP. https://cdn.ymaws.com/npiap.com/resource/resmgr/white_papers/Unavoidable_in_COVID_Pandemi.pdf.

[10] Tang J., Li B., Gong J., Li W., Yang J. (2020). Challenges in the management of critical ill COVID-19 patients with pressure ulcer. Int. Wound J..

[11] Kuniavsky M., Vilenchik E., Lubanetz A. (2020). Under (less) pressure—Facial pressure ulcer development in ventilated ICU patients: A prospective comparative study comparing two types of endotracheal tube fixations. Intensive Crit. Care Nurs..

[12] De Ligt M., Hesselink M.K.C., Jorgensen J., Hoebers N., Blaak E.E., Goossens G.H. (2021). Resveratrol supplementation reduces ACE2 expression in human adipose tissue. Adipocyte.

[13] Al-Benna S. (2020). Association of high level gene expression of ACE2 in adipose tissue with mortality of COVID-19 infection in obese patients. Obes. Med..

[14] Scheja L., Heeren J. (2019). The endocrine function of adipose tissues in health and cardiometabolic disease. Nat. Rev. Endocrinol..

[15] Poma A.M., Bonuccelli D., Giannini R., Macerola E., Vignali P., Ugolini C., Torregrossa L., Proietti A., Pistello M., Basolo A. (2021). COVID-19 autopsy cases: Detection of virus in endocrine tissues. J. Endocrinol. Investig..

[16] Yu N., Zhang Y., Xiao M., Cao W., Zhang Y., Yang Y., Liu Z., Li Y., Long X., Liu Z. (2021). SARS-CoV-2 not found in pressure injury exudates from COVID-19 patients. J. Cosmet. Dermatol..

[17] McNally A.K., Anderson J.M. (2011). Macrophage fusion and multinucleated giant cells of inflammation. Adv. Exp. Med. Biol..

[18] Imai Y., Ibata I., Ito D., Ohsawa K., Kohsaka S. (1996). A Novel Geneiba1in the Major Histocompatibility Complex Class III Region Encoding an EF Hand Protein Expressed in a Monocytic Lineage. Biochem. Biophys. Res. Commun..

[19] Knisely A., Zhou Z.N., Wu J., Huang Y., Holcomb K., Melamed A., Advincula A.P., Lalwani A., Khoury-Collado F., Tergas A.I. (2021). Perioperative morbidity and mortality of patients with COVID-19 who undergo urgent and emergent surgical procedures. Ann. Surg..

[20] Blum H.E., Deus B., Gerok W. (1983). Mitochondrial creatine kinase from human heart muscle: Purification and characterization of the crystallized isoenzyme. J. Biochem..

[21] Mao L., Jin H., Wang M., Hu Y., Chen S., He Q., Chang J., Hong C., Zhou Y., Wang D. (2020). Neurologic Manifestations of Hospitalized Patients with Coronavirus Disease 2019 in Wuhan, China. JAMA Neurol..

[22] Akbar M.R., Pranata R., Wibowo A., Lim M.A., Sihite T.A., Martha J.W. (2021). The prognostic value of elevated creatine kinase to predict poor outcome in patients with COVID-19—A systematic review and meta-analysis: Creatinine Kinase in COVID-19. Diabetes Metab. Syndr. Clin. Res. Rev..

[23] Orsucci D., Trezzi M., Anichini R., Blanc P., Barontini L., Biagini C., Capitanini A., Comeglio M., Corsini P., Gemignani F. (2021). Increased creatine kinase may predict a worse covid-19 outcome. J. Clin. Med..

[24] Hunter T. (2000). Signaling—2000 and Beyond. Cell.

[25] Besant P., Attwood P., Piggott M. (2009). Focus on Phosphoarginine and Phospholysine. Curr. Protein Pept. Sci..

[26] Brooks P.J., Glogauer M., McCulloch C.A. (2019). An Overview of the Derivation and Function of Multinucleated Giant Cells and Their Role in Pathologic Processes. Am. J. Pathol..

[27] Lê K.-A., Mahurkar S., Alderete T.L., Hasson R.E., Adam T.C., Kim J.S., Beale E., Xie C., Greenberg A.S., Allayee H. (2011). Subcutaneous Adipose Tissue Macrophage Infiltration Is Associated with Hepatic and Visceral Fat Deposition, Hyperinsulinemia, and Stimulation of NF-κB Stress Pathway. Diabetes.

[28] Braune J., Lindhorst A., Fröba J., Hobusch C., Kovacs P., Blüher M., Eilers J., Bechmann I., Gericke M. (2021). Multinucleated giant cells in adipose tissue are specialized in adipocyte degradation. Diabetes.

[29] Huang Z.H., Manickam B., Ryvkin V., Zhou X.J., Fantuzzi G., Mazzone T., Sam S. (2013). PCOS Is Associated with Increased CD11c Expression and Crown-Like Structures in Adipose Tissue and Increased Central Abdominal Fat Depots Independent of Obesity. J. Clin. Endocrinol. Metab..

[30] Apovian C.M., Bigornia S., Mott M., Meyers M.R., Ulloor J., Gagua M., McDonnell M., Hess D., Joseph L., Gokce N. (2008). Adipose Macrophage Infiltration Is Associated with Insulin Resistance and Vascular Endothelial Dysfunction in Obese Subjects. Arterioscler. Thromb. Vasc. Biol..

[31] Miron R.J., Bosshardt D.D. (2018). Multinucleated Giant Cells: Good Guys or Bad Guys?. Tissue Eng.-Part B Rev..

[32] Suess C., Hausmann R. (2020). Gross and histopathological pulmonary findings in a COVID-19 associated death during self-isolation. Int. J. Legal Med..

[33] Pernazza A., Mancini M., Rullo E., Bassi M., De Giacomo T., Della Rocca C., d’Amati G. (2020). Early histologic findings of pulmonary SARS-CoV-2 infection detected in a surgical specimen. Virchows Arch..

[34] Oprinca G.C., Muja L.A. (2021). Postmortem examination of three SARS-CoV-2-positive autopsies including histopathologic and immunohistochemical analysis. Int. J. Legal Med..

[35] Vishwajeet V., Purohit A., Kumar D., Vijayvergia P., Tripathi S., Kanchan T., Kothari N., Dutt N., Elhence P.A., Bhatia P.K. (2021). Evaluation of Liver Histopathological Findings of Coronavirus Disease 2019 by Minimally Invasive Autopsies. J. Clin. Exp. Hepatol..

[36] Xu Z., Shi L., Wang Y., Zhang J., Huang L., Zhang C., Liu S., Zhao P., Liu H., Zhu L. (2020). Pathological findings of COVID-19 associated with acute respiratory distress syndrome. Lancet Respir. Med..

[37] Venter G., Polling S., Pluk H., Venselaar H., Wijers M., Willemse M., Fransen J.A.M., Wieringa B. (2015). Submembranous recruitment of creatine kinase B supports formation of dynamic actin-based protrusions of macrophages and relies on its C-terminal flexible loop. Eur. J. Cell Biol..

[38] Loike J.D., Kozler V.F., Silverstein S.C. (1984). Creatine kinase expression and creatine phosphate accumulation are developmentally regulated during differentiation of mouse and human monocytes. J. Exp. Med..

[39] Gagiannis D., Steinestel J., Hackenbroch C., Schreiner B., Hannemann M., Bloch W., Umathum V.G., Gebauer N., Rother C., Stahl M. (2020). Clinical, Serological, and Histopathological Similarities Between Severe COVID-19 and Acute Exacerbation of Connective Tissue Disease-Associated Interstitial Lung Disease (CTD-ILD). Front. Immunol..

[40] Wang E.Y., Mao T., Klein J., Dai Y., Huck J.D., Jaycox J.R., Liu F., Zhou T., Israelow B., Wong P. (2021). Diverse functional autoantibodies in patients with COVID-19. Nature.

[41] Abdi A., AlOtaiby S., Al Badarin F., Khraibi A., Hamdan H., Nader M. (2022). Interaction of SARS-CoV-2 with cardiomyocytes: Insight into the underlying molecular mechanisms of cardiac injury and pharmacotherapy. Biomed. Pharmacother..

[42] Shevchenko A., Wilm M., Vorm O., Mann M. (1996). Mass spectrometric sequencing of proteins from silver-stained polyacrylamide gels. Anal. Chem..

[43] Hughes C.S., Foehr S., Garfield D.A., Furlong E.E., Steinmetz L.M., Krijgsveld J. (2014). Ultrasensitive proteome analysis using paramagnetic bead technology. Mol. Syst. Biol..

[44] Shilov I.V., Seymour S.L., Patel A.A., Loboda A., Tang W.H., Keating S.P., Schaeffer D.A. (2007). The paragon algorithm, a next generation search engine that uses sequence temperature values sequence temperature values and feature probabilities to identify peptides from tandem mass spectra. Mol. Cell. Proteom..

[45] Arevalo-Martin A., Grassner L., Garcia-Ovejero D., Paniagua-Torija B., Barroso-Garcia G., Arandilla A.G., Molina-Holgado E. (2018). Elevated autoantibodies in subacute human spinal cord injury are naturally occurring antibodies. Front. Immunol..

