# Supplementary material for: Post-COVID Complications after Pressure Ulcer Surgery in Patients with Spinal Cord Injury Associate with Creatine Kinase Upregulation in Adipose Tissue
Source: Cells. 2022 Apr 9;11(8):1282. doi: 10.3390/cells11081282 (PMC9025224; doi:10.3390/cells11081282)
Supplement: Supplementary file 1 [file cells-11-01282-s001.zip › cells-1615019-supplementary.pdf]

Supplementay materials

1. Proteomic analysis

1.1. 2D-Gel based proteomics

Protein samples (150 µg) were precipitated using the 2D Clean-up kit (GE Healthcare Bio-Sciences Corp), following manufacture's protocol, and then resuspended in rehydration solution containing 7 M urea, 2 M thiourea, 4% CHAPS, 30 Mm Tris, 1% DTT 2 M, 1.2% DeStreak Reagent (GE Healthcare) and 2% IPG buffer (pH 3-11NL, GE Healthcare), to a final volume of 200 µL. Samples were loaded onto the IPG strips (11 cm, pH 3-11NL) by 12 h active rehydration at 50 V and 20 ºC. Each IPG strip was subjected to IEF in the PROTEAN i12 IEF Cell, according to the following protocol: 500 V for 30 min, 1000 V for 1 h, linear ramp to 5000 V over 3 h followed by 5000 for 7 hr.

A current limit of 50 µA was held throughout. After IEF, the strips were equilibrated with 1% DTT in equilibration buffer (1.5 M Tris, pH 8.8 buffer containing 6 M urea, 30% glycerol, 2% SDS and bromophenol blue), for 20 min, followed by incubation with 2.5% iodoacetamide in the same buffer for 20 min. SDS-PAGE was performed 1 h 30min, using the Criterion Cell Electrophoresis System (Bio-Rad), at 15 W/gel. Gels were stained with Page Blue Protein Staining solution (Thermo Scientific). Coomassie stained gels were recorded as digitalized images using an Image Scanner III (GE Healthcare).

1.2. Mass spectrometry

Spots from 2D-WB were excised and digested according to Schevchenko *et al*. [1] with minor modifications: gel cuts were incubated with 10 mM DTT in 50mM ammonium bicarbonate (Ambic), for 60min at 37ºC and after reduction, alkylation with 55mM iodoacetamide in 50mM Ambic was carried out for 20min at RT. Gel plugs were washed with 50mM Ambic in 50% methanol, rinsed in acetonitrile (ACN) and dried in a Speedvac. Dry gel pieces were then embedded in sequencing grade modified porcine trypsin (Promega, Madison, WI, USA) at a final concentration of 12.5ng/µL in 20 mM Ambic. After digestion at 37 ºC overnight, peptides were extracted with 0.5% formic acid (FA) in 60% ACN, dried in sped-vac and resuspended in 2% FA, 2% ACN.

Peptide samples were analyzed with by MALDI-MS/MS, using a 4800 Plus MALDI TOF/TOF Analyzer (Applied Biosystems). Spectra were acquired in the reflector positive-ion mode with a Nd:YAG, 355nm wavelength laser, at 200 Hz laser frequency, and 1000 to 2000 individual spectra were averaged. For MS/MS 1kV analysis mode, precursors were accelerated to 8 kV in source 1, selected with a relative resolution of 200 (FWHM) and metastable suppression. Fragment ions generated by collision with air in a CID chamber were further accelerated by 15 kV in source 2. Automated analysis of mass data was performed using the 4000 Series Explorer Software version 3.7.0 (Applied Biosystems). Internal calibration of MALDI-TOF mass spectra was performed using two trypsin autolysis ions with m/z: 842.510 and 2211.105 Da. For MALDI-MS/MS, calibrations were performed with fragment ion spectra obtained for Glub-fibrinopeptide (4700 Cal Mix, Applied Biosystems).

Peptide and protein identifications were performed using ProteinPilotTM Software V 5.0 (Sciex) using MASCOT Versions 2.5 (Matrix Science Ltd., UK) against the SwissProt Fasta dataset (2021 May, 564,277 sequences; 203340877 residues). Parameters applied: 1 trypsin miss-cleavage; methionine oxidation (variable modification), cysteine carbamidomethylation (fixed modification); precursor mass tolerance of 50 ppm; MS/MS tolerance of 0.6 amu; significance threshold (p) below 0.05 (MudPIT scoring) and minimal protein Mascot score of 56.

1.3. SWATH

Samples lysates were digested using Single-pot solid-phase-enhanced sample preparation (SP3) according to Hughes et al [2]. Samples were reduced and alkylated using DTT and IAA, respectively and then incubated with 6 µL of the prepared bead mix, in 50 µL final volume ofH_2O. Afterward, EtOH was added to a final concentration of 50% (v/v) and samples were left stirring at 1000 rpm and room temperature for 5 min. Subsequently, beads were immobilized by incubation on a magnetic rack for 2 min. The supernatant was discarded and the pellet was rinsed with 80% (v/v) EtOH in water. Beads were resuspended in 300 µl of 100 mM NH_4HCO_3 supplemented with trypsin/LysC in an enzyme to protein ratio of 1:25 (w/w). After digestion overnight at 37 °C and 1000 rpm, samples were centrifuged at 20,000 g, and the supernatant was collected and acidified using 2% FA.

Samples were pooled and 3 µg were separated into a Ekspert™ nanoLC425 (Eksigent, Dublin, CA, USA) using a C18 column (ChromXPC18, 3µm, 120Å 0.075 x 150 mm, Eksigent) at a flow rate of 300 nL/min in combination with a precolumn (NanoLC Trap ChromXP C18, 3µm 120Å, Eksigent) at a flow rate of 5 µL/min. The buffers used were: A (0.1% FA 2% ACN) and B (0.1% FA, 98% ACN)). Peptides were desalted for 3 min with 0.1% FA/2% ACN on the precolumn, followed by 85 min gradient from 5% to 30% of solvent B, 30%-95% for 0.1 min, and finally 95% of solvent B for 5 min.

Peptides eluted were directly injected into a hybrid quadrupole-TOF mass spectrometer TripleTOF® 6600+ (Sciex, Redwood City, CA, USA). Samples were ionized in a source type Optiflow < 1µL Nano applying 3.0 kV to the spray emitter at 200 °C. Analysis was carried out in a data-dependent positive ion mode (DDA). Survey MS1 scans were acquired 350-1400 m/z for 250 ms. The quadrupole resolution was set to "LOW" for MS2 experiments, which were acquired 100-1500 m/z for 25 ms in "high sensitivity" mode. Following switch criteria were used: charge 2+ to 4+; minimum intensity 250 counts per second (cps). Up to 100 ions were selected for fragmentation after each survey scan. Dynamic exclusion was set at 15 s.

The TripleTOF was operated in SWATH mode, in which a 50 ms TOF MS scan from 350–1250 m/z was performed, followed by 80 ms product ion scans from 350–1250 m/z on the 37 defined windows of 15 Da window widths from 450 to 1000 Da (3.05 sec/cycle). The individual SWATH injections were randomized.

Peptide and protein identifications were performed using ProteinPilot™ Software V 5.0 (Sciex) and the Paragon algorithm [3]. Each MS/MS spectrum was searched against the SwissProt-SARS2 database, with the fixed modification of carbamidomethyl -labelled cysteine parameter enabled. A False Discovery rate (FDR) of 1% was used.

SWATH Acquisition MicroApp v.2.0 was used for data processing and building a peptide spectral library containing the peptides identified in the database search with confidence scores above 99%. Parameters used: 20 peptides/protein; 6 fragment ions/peptide; extraction windows of 5 min and 20 ppm; peptide FDR of 1% and confidence score threshold of 95%.

Normalization of the protein abundance signal as measured by SWATH was carried out using MarkerView (v1.2.1, Sciex), and testing for differential abundance was performed at the protein level by applying a Student's t-test.

2. Western Blot (WB)

Tissue extracts were mixed with 2x Laemmli sample buffer (Tris HCl 25mM pH 6.8, 10 % glycerol, 6 % SDS, 0.1 % bromophenol blue) supplemented with 50 mM DTT and 0.5 M β-mercaptoethanol and heated at 37 °C for 10 min, before proteins (10–100 μg) were resolved on 10 % SDS- polyacrylamide gels. Page ruler pre-stained protein ladders were used as molecular weight standards (ThermoFisher Scientific 26616, 26619). After electroblotting, the nitrocellulose membranes were blocked for 1 h at RT in 5 % (w/v) blotting grade non-fat dry milk in TBS plus 0.1 % Tween-20. Membranes were then probed overnight at 4 °C with different antibodies: Anti-SARS-CoV-2 spike glycoprotein antibody (ab272504) 1:1000 dilution; SARS-CoV / SARS-CoV-2 (COVID-19) spike antibody [1A9] (GTX632604) 1/2000 dilution; SARS-CoV-2 (COVID-19) nucleocapsid antibody (GTX 135357) 1/5000 dilution; Anti-Creatine Kinase MM antibody (ab151465) 1:2000 dilution; Purified anti-CKMT2 Antibody (Biolegend; 868702) 6.5/1000 dilution; Purified Mouse Anti-Phosphoserine/threonine (BD Biosciences, 612549) 1/2500 dilution. These antibodies are referred to as spike, nucleocapside, CKM, CKMT2, and Ser/The respectively, throughout the text. Antibodies against GAPDH (ab8245, Abcam PLC, Cambridge, UK; RRIDI: AB_2107448); 1:2×105; 1:50000 were used to determine total protein loading. Subsequently, the blots were incubated with horseradish peroxidase-conjugated secondary antibodies (Jackson immunoResearch) and visualized by chemiluminescence using SuperSignal West Pico Substrate Detection Kit (ThermoFisher Scientific 34580) in a GE 6000 image analyzer. When necessary, the blots were stripped with Restore™ Western Blot Stripping Buffer (ThermoFisher Scientific 21059), for 20 min at 55 °C and then re-probed as indicated in the text. The optical density of protein labeling was quantified using ImageJ software (NIH, Bethesda, Maryland, USA). Additionally, secondary antibodies conjugated with 680 or 800 infrared fluorophores were used for the simultaneous analysis of CKM and Gapdh in separate fluorescent channels using the Odyssey Infrared Imaging System (LICOR). Relative quantification of CKM using GADPH as an internal loading control was carried out following the Odyssey LI-COR manual, and was normalized against the value of the CKM/GADPH value found in the B0 sample considered as 100%.

3. RT-PCR SARS-CoV-2

Adipose tissue (50-100 mg) was cut into small pieces and homogenized with a power homogenizer in 1 mL of QIAzol lysis reagent (Qiagen Sciences, Madrid, Spain). Total RNA purification was carried out by chloroform extraction and isopropanol precipitation. RNA was retrotranscribed into cDNA with the High-capacity RNA-to-cDNA kit (4387406, Thermo Fisher Scientific) following the manufacturer´s

instructions. For qRTPCR the same amount of cDNA from each sample was used (100 ng) and specific Taqman probes were used for the SARS-CoV-2 S gene (Vi07918636_s1) and the 18S gene used as an internal load control (Hs99999901_s1; Thermo Fisher Scientific). PCR reactions were performed in a 7900HT Fast Real-Time PCR System (Applied Biosystems) and automatic detection of Ct was used to establish the threshold of amplification for each gene.

4. **Histology and immunohistochemistry**

Biopsies were immersed in 10% formalin solution for 24h at 4°C. Tissue was then dehydrated in grading ethanol solutions, cleared with xylene and transferred to consecutive solutions of freshly prepared paraffin (60°C) until finally embedded. Paraffin embedded samples were maintained at room temperature and protected from light until used. Ten m thick sections were then obtained with a Leica RM2265 Microtome (Leica Biosystems, Barcelona, Spain) and collected in Superfrost Plus slides (Menzel Glaser, Thermo Scientific).

4.1. *Hematoxylin and Eosin staining:*

Tissue was dewaxed in xylene (3 x 5 min) and rehydrated in a decreasing gradient of EtOH (3 x 5 min 100%, 2 x 3 min 96%, 1 x 3min 70%). Sections were then stained with Harris Haematoxilin (5min), rinsed in tap water and differentiated in 1% HCl in 70% ethanol. After further rinsing, slices were counterstained with eosin (5 min), dehydrated and mounted.

4.2. *Immunohistochemistry:*

Sections were deparaffinated as described above and rinsed in 0.1 M PBS. Endogenous peroxidase activity was inhibited by incubating the sections in 0.1 M PBS containing 1.8% H_2O_2 and 50% methanol. Sections were rinsed extensively with rinse solution RS (PBS 0.1M + 0.3% bovine serum albumin –BSA- plus 0.01% Triton X-100). The sections were then subjected to antigen unmasking, consisting of a 20 min pretreatment with a 10 mM sodium citrate solution, pH: 6, 0.01% Triton X-100, at 55 C. After extensive rinsing with RS, sections were incubated for 48 h with the following primary antibodies diluted in RS and 5% BSA: monoclonal mouse IgG2b anti-CKMT2 (1:250), rabbit anti CKMM (1:500) or rabbit anti Iba1 (Wako; #019-19741, 1:500). Sections were then rinsed and incubated for 90 minutes at RT with a biotin-conjugated secondary antibody (1:500; Jackson Immunoresearch Europe, Ely, Cambridgeshire, United Kingdom) rinsed again and finally incubated with Horseradish Peroxidase (HRP)-conjugated streptavidin (1:500; Jackson Immunoresearch Europe, Ely, Cambridgeshire, United Kingdom), except for Iba1, that was detected with a HRP-conjugated Donkey anti-rabbit antibody (1:500; Jackson Immunoresearch Europe, Ely, Cambridgeshire, United Kingdom). A brown colour was developed using a solution of 0.03% diaminobenzidine and 0.01% hydrogen peroxide in 0.1 M PBS, after which sections were mildly counterstained with toluidine blue to visualise cell nuclei, dehydrated and mounted. Samples were analysed and photographed using an Olympus BX61 microscope with an Olympus DP71 digital camera attached (Olympus Iberia, Barcelona, Spain) at the Microscope Facilities in the Hospital Nacional de Parapléjicos (Toledo, Spain).

5. **Detection of human autoantibodies by two dimensional-western blot**

Detection of human autoantibodies was performed according to Arevalo-Martin et al [4], with minor modifications. Briefly, we made two groups, one group with pre-pandemic SCI patient samples and another with post-COVID-19 patient samples. Proteins extracted from the tissues of each group were pooled. Proteins were precipitated using the 2D Clean Up Kit (Sigma, Madrid, Spain) following manufacturer's instructions and resuspended in labeling buffer containing 7 M urea, 2 M thiourea, 4% CHAPS and 30 mM Tris. After checking samples to be between pH 8.0–9.0, soluble and insoluble fractions were mixed in a 1:1 ratio. Samples were then resolved in a 2D-EF gel, proceeding as described above, and then proteins were transferred to low fluorescence PVDF membranes with a semidry protocol using Trans-Blot® Turbo™ transfer system (Bio-Rad).

Membranes were blocked with BlockAid solution (Thermo Fisher Scientific, Madrid, Spain) for 30 min at RT, and incubated overnight at 4°C with patient or healthy subjects sera diluted 1:250 in PBS, 0.1% Triton X-100 and 10% BlockAid Solution. After several washing steps, membranes were incubated for 2 h at RT with secondary Cy3-conjugated goat anti-human IgG (Fcγ fragment specific; 1:1,000; Jackson Immunoresearch) and Alexa-647 goat anti-human IgM (Fc5μ fragment specific; 1:1,000; Jackson Immunoresearch). 2D-WB were visualized with the laser confocal scanner Typhoon ™ Trio (GE Healthcare) and images were acquired at 100 μm / pixel with a 16-bit depth.

2D-Western-blots were performed with serum from post-COVID-19 patients and serum from volunteers obtained before the start of the pandemic (pre-pandemic < 2016). The reproducibility of the procedure was confirmed after finding similar bound Ab levels in four replicates for each sample.

References

1. Shevchenko, A.; Wilm, M.; Vorm, O.; Mann, M. Mass spectrometric sequencing of proteins from silver-stained polyacrylamide gels. *Anal. Chem.* **1996**, *68*, 850–858. https://doi.org/10.1021/ac950914h.
2. Hughes, C.S.; Foehr, S.; Garfield, D.A.; Furlong, E.E.; Steinmetz, L.M.; Krijgsveld, J. Ultrasensitive proteome analysis using paramagnetic bead technology. *Mol. Syst. Biol.***2014**, *10*, 757. https://doi.org/10.15252/msb.20145625.
3. Shilov, I.V.; Seymour, S.L.; Patel, A.A.; Loboda, A.; Tang, W.H.; Keating, S.P.; Schaeffer, D.A. The paragon algorithm, a next generation search engine that uses sequence temperature values sequence temperature values and feature probabilities to identify peptides from tandem mass spectra. *Mol. Cell. Proteom.* **2007**, *6*, 1638–1655. https://doi.org/10.1074/mcp.T600050-MCP200.
4. Arevalo-Martin, A.; Grassner, L.; Garcia-Ovejero, D.; Paniagua-Torija, B.; Barroso-Garcia, G.; Arandilla, A.G.; ; Molina-Holgado, E. Elevated autoantibodies in subacute human spinal cord injury are naturally occurring antibodies. *Front. Immunol.* **2018**, *9*, 2365. https://doi.org/10.3389/fimmu.2018.02365.

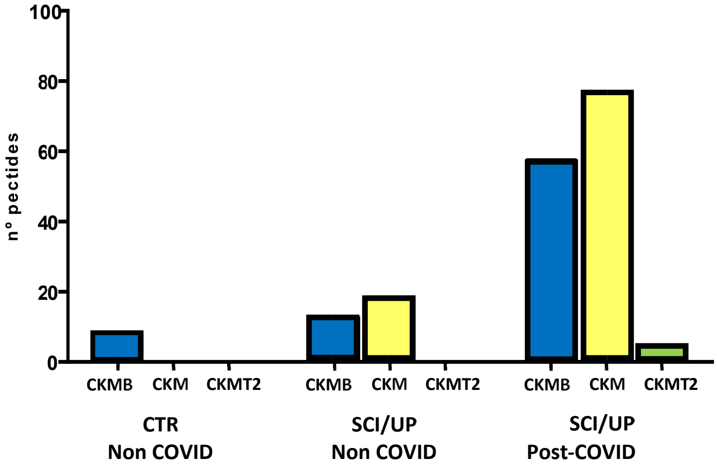

Supplementary Figure S1. Analysis of the total protein content of each group by LC-MS/MS. For this analysis we include a new control group of SARS-CoV-2 negative patients without SCI or PU CTR (non-COVID). The graph shows the number of peptides identified corresponding to three different creatine kinase isoforms: CKMB, CKM, and CKMT2.

Supplementary Table S1: Clinical characteristics of the patients recruited in the control group, without SCI and PU**.** Control group (CTR NON-COVID, Without SCI/PU). Abbreviations: (SCI): Spinal Cord Injury; (PU): Pressure Ulcers; (M): Man; (W): woman; (COVID): Coronavirus disease.

	Subjects	Age	Sex	Surgical intervention	Comorbility
CTR WITHOUT SCI / PU NON-COVID	1	55	M	knee arthroplasty	Dyslipidemia, Arterial Hypertension
	2	60	M	knee arthroplasty	Not known
	3	28	M	Tibial osteosynthesis	Not known
	4	69	W	Prosthetic Replacement	Dyslipidemia, Arterial Hypertension
	5	47	W	Hip arthroplasty	Arterial hypertension, Diabetic
	6	61	M	Discectomy and l4 l5 arthrodesis	Diabetic Dyslipidemia, Arterial Hypertension
	7	74	M	Aortic valve replacement	Dyslipidemia, Arterial Hypertension
	8	71	M	knee arthroplasty	Arterial Hypertension
	9	34	W	l4 l5 arthrodesis	Not known
	10	61	W	Lipoma Excision	Diabetic Dyslipidemia, Arterial Hypertension

Supplementary Table S2. List of proteins identified in the three spots selected from the 2D-gels of adipose tissue from non-COVID and postCOVID patients. The table includes de following parameters: **N**: rack of a particular protein in respect to the other proteins identified; **%Cov(95)**: the number of amino acids matching the identified protein sequence with confidence greater than 95 %, divided by the total number of amino acids in the protein; **Accession**: Identification number for the protein from the squid protein sequence database; **Name**: Protein sequence name and taxonomic homology; **Peptides:** number of peptides identified

N	%Cov(95)	Accession	Name	Peptides
**Non-COVID_Spot 1 (Proteins)**				
6	51,050	sp|Q9BXN1|ASPN_HUMAN	Asporin OS=Homo sapiens OX=9606 GN=ASPN PE=1 SV=2	19
7	15,060	sp|P42704|LPPRC_HUMAN	Leucine-rich PPR motif-containing protein, mitochondrial OS=Homo sapiens OX=9606 GN=LRPPRC PE=1 SV=3	19
8	23,660	sp|P06396|GELS_HUMAN	Gelsolin OS=Homo sapiens OX=9606 GN=GSN PE=1 SV=1	19
4	47,830	sp|P49753|ACOT2_HUMAN	Acyl-coenzyme A thioesterase 2, mitochondrial OS=Homo sapiens OX=9606 GN=ACOT2 PE=1 SV=6	17
11	16,050	sp|P02671|FIBA_HUMAN	Fibrinogen alpha chain OS=Homo sapiens OX=9606 GN=FGA PE=1 SV=2	15
9	37,830	sp|P49411|EFTU_HUMAN	Elongation factor Tu, mitochondrial OS=Homo sapiens OX=9606 GN=TUFM PE=1 SV=2	13
10	38,890	sp|O75874|IDHC_HUMAN	Isocitrate dehydrogenase [NADP] cytoplasmic OS=Homo sapiens OX=9606 GN=IDH1 PE=1 SV=2	13
12	51,330	sp|P61163|ACTZ_HUMAN	Alpha-centractin OS=Homo sapiens OX=9606 GN=ACTR1A PE=1 SV=1	12
13	33,020	sp|P11310|ACADM_HUMAN	Medium-chain specific acyl-CoA dehydrogenase, mitochondrial OS=Homo sapiens OX=9606 GN=ACADM PE=1 SV=1	11
14	35,800	sp|P16930|FAAA_HUMAN	Fumarylacetoacetase OS=Homo sapiens OX=9606 GN=FAH PE=1 SV=2	10
15	28,610	sp|P06732|KCRM_HUMAN	**Creatine kinase M-type** OS=Homo sapiens OX=9606 GN=CKM PE=1 SV=2	10
16	28,090	sp|P50395|GDIB_HUMAN	Rab GDP dissociation inhibitor beta OS=Homo sapiens OX=9606 GN=GDI2 PE=1 SV=2	9

19	33,330	sp|P00558|PGK1_HUMAN	Phosphoglycerate kinase 1 OS=Homo sapiens OX=9606 GN=PGK1 PE=1 SV=3	9
20	15,190	sp|P33121|ACSL1_HUMAN	Long-chain-fatty-acid--CoA ligase 1 OS=Homo sapiens OX=9606 GN=ACSL1 PE=1 SV=1	9
21	36,010	sp|Q86VN1|VPS36_HUMAN	Vacuolar protein-sorting-associated protein 36 OS=Homo sapiens OX=9606 GN=VPS36 PE=1 SV=1	9
22	29,950	sp|P09972|ALDOC_HUMAN	Fructose-bisphosphate aldolase C OS=Homo sapiens OX=9606 GN=ALDOC PE=1 SV=2	9
23	45,880	sp|P04075|ALDOA_HUMAN	Fructose-bisphosphate aldolase A OS=Homo sapiens OX=9606 GN=ALDOA PE=1 SV=2	9
17	25,470	sp|P52209|6PGD_HUMAN	6-phosphogluconate dehydrogenase, decarboxylating OS=Homo sapiens OX=9606 GN=PGD PE=1 SV=3	8
18	70,750	sp|P68871|HBB_HUMAN	Hemoglobin subunit beta OS=Homo sapiens OX=9606 GN=HBB PE=1 SV=2	8
24	27,950	sp|P08559|ODPA_HUMAN	Pyruvate dehydrogenase E1 component subunit alpha, somatic form, mitochondrial OS=Homo sapiens OX=9606 GN=PDHA1 PE=1 SV=3	8
25	32,240	sp|P42765|THIM_HUMAN	3-ketoacyl-CoA thiolase, mitochondrial OS=Homo sapiens OX=9606 GN=ACAA2 PE=1 SV=2	8
27	28,060	sp|P0DOX5|IGG1_HUMAN	Immunoglobulin gamma-1 heavy chain OS=Homo sapiens OX=9606 PE=1 SV=2	7
26	24,630	sp|P62195|PRS8_HUMAN	26S proteasome regulatory subunit 8 OS=Homo sapiens OX=9606 GN=PSMC5 PE=1 SV=1	6
31	18,040	sp|Q92947|GCDH_HUMAN	Glutaryl-CoA dehydrogenase, mitochondrial OS=Homo sapiens OX=9606 GN=GCDH PE=1 SV=1	6
29	18,400	sp|P63261|ACTG_HUMAN	Actin, cytoplasmic 2 OS=Homo sapiens OX=9606 GN=ACTG1 PE=1 SV=1	5
30	15,790	sp|Q96DV4|RM38_HUMAN	39S ribosomal protein L38, mitochondrial OS=Homo sapiens OX=9606 GN=MRPL38 PE=1 SV=2	5
32	15,070	sp|P36955|PEDF_HUMAN	Pigment epithelium-derived factor OS=Homo sapiens OX=9606 GN=SERPINF1 PE=1 SV=4	5
33	17,740	sp|Q99447|PCY2_HUMAN	Ethanolamine-phosphate cytidylyltransferase OS=Homo sapiens OX=9606 GN=PCYT2 PE=1 SV=1	5

34	20,110	sp|P21810|PGS1_HUMAN	Biglycan OS=Homo sapiens OX=9606 GN=BGN PE=1 SV=2	5
35	26,440	sp|P00915|CAH1_HUMAN	Carbonic anhydrase 1 OS=Homo sapiens OX=9606 GN=CA1 PE=1 SV=2	5
36	19,940	sp|P01859|IGHG2_HUMAN	Immunoglobulin heavy constant gamma 2 OS=Homo sapiens OX=9606 GN=IGHG2 PE=1 SV=2	5
28	13,870	sp|P51888|PRELP_HUMAN	Prolargin OS=Homo sapiens OX=9606 GN=PRELP PE=1 SV=1	4
37	4,244	sp|P11498|PYC_HUMAN	Pyruvate carboxylase, mitochondrial OS=Homo sapiens OX=9606 GN=PC PE=1 SV=2	4
39	9,108	sp|P04040|CATA_HUMAN	Catalase OS=Homo sapiens OX=9606 GN=CAT PE=1 SV=3	4
42	28,170	sp|P69905|HBA_HUMAN	Hemoglobin subunit alpha OS=Homo sapiens OX=9606 GN=HBA1 PE=1 SV=2	4
54	12,150	sp|P07477|TRY1_HUMAN	Trypsin-1 OS=Homo sapiens OX=9606 GN=PRSS1 PE=1 SV=1	4
38	10,360	sp|P30533|AMRP_HUMAN	Alpha-2-macroglobulin receptor-associated protein OS=Homo sapiens OX=9606 GN=LRPAP1 PE=1 SV=1	3
40	8,096	sp|Q9Y697|NFS1_HUMAN	Cysteine desulfurase, mitochondrial OS=Homo sapiens OX=9606 GN=NFS1 PE=1 SV=3	3
43	7,296	sp|O75390|CISY_HUMAN	Citrate synthase, mitochondrial OS=Homo sapiens OX=9606 GN=CS PE=1 SV=2	3
44	8,571	sp|Q96IJ6|GMPPA_HUMAN	Mannose-1-phosphate guanyltransferase alpha OS=Homo sapiens OX=9606 GN=GMPPA PE=1 SV=1	3
45	12,030	sp|Q8WTS1|ABHD5_HUMAN	1-acylglycerol-3-phosphate O-acyltransferase ABHD5 OS=Homo sapiens OX=9606 GN=ABHD5 PE=1 SV=1	3
46	9,223	sp|P00966|ASSY_HUMAN	Argininosuccinate synthase OS=Homo sapiens OX=9606 GN=ASS1 PE=1 SV=2	3
47	7,662	sp|P09622|DLDH_HUMAN	Dihydrolipoyl dehydrogenase, mitochondrial OS=Homo sapiens OX=9606 GN=DLD PE=1 SV=2	3
48	5,164	sp|P26440|IVD_HUMAN	Isovaleryl-CoA dehydrogenase, mitochondrial OS=Homo sapiens OX=9606 GN=IVD PE=1 SV=2	3
50	9,014	sp|Q14103|HNRPD_HUMAN	Heterogeneous nuclear ribonucleoprotein D0 OS=Homo sapiens OX=9606 GN=HNRNPD PE=1 SV=1	3
51	10,610	sp|Q9NTK5|OLA1_HUMAN	Obg-like ATPase 1 OS=Homo sapiens OX=9606 GN=OLA1 PE=1 SV=2	3
55	6,024	sp|P02545|LMNA_HUMAN	Prelamin-A/C OS=Homo sapiens OX=9606 GN=LMNA PE=1 SV=1	3

52	3,590	sp|Q99798|ACON_HUMAN	Aconitate hydratase, mitochondrial OS=Homo sapiens OX=9606 GN=ACO2 PE=1 SV=2	2
53	7,576	sp|Q5VTE0|EF1A3_HUMAN	Putative elongation factor 1-alpha-like 3 OS=Homo sapiens OX=9606 GN=EEF1A1P5 PE=5 SV=1	2
56	6,684	sp|P11766|ADHX_HUMAN	Alcohol dehydrogenase class-3 OS=Homo sapiens OX=9606 GN=ADH5 PE=1 SV=4	2
57	7,507	sp|P15104|GLNA_HUMAN	Glutamine synthetase OS=Homo sapiens OX=9606 GN=GLUL PE=1 SV=4	2
58	1,274	sp|P49327|FAS_HUMAN	Fatty acid synthase OS=Homo sapiens OX=9606 GN=FASN PE=1 SV=3	2
60	6,094	sp|P08397|HEM3_HUMAN	Porphobilinogen deaminase OS=Homo sapiens OX=9606 GN=HMBS PE=1 SV=2	2
61	6,623	sp|P22695|QCR2_HUMAN	Cytochrome b-c1 complex subunit 2, mitochondrial OS=Homo sapiens OX=9606 GN=UQCRC2 PE=1 SV=3	2
63	0,945	sp|Q9Y490|TLN1_HUMAN	Talin-1 OS=Homo sapiens OX=9606 GN=TLN1 PE=1 SV=3	2
65	4,429	sp|Q9NVA2|SEP11_HUMAN	Septin-11 OS=Homo sapiens OX=9606 GN=SEPTIN11 PE=1 SV=3	2
**Non-COVID_Spot 2 (Proteins)**				
4	66,050	sp|P49753|ACOT2_HUMAN	Acyl-coenzyme A thioesterase 2, mitochondrial OS=Homo sapiens OX=9606 GN=ACOT2 PE=1 SV=6	29
10	48,550	sp|O75874|IDHC_HUMAN	Isocitrate dehydrogenase [NADP] cytoplasmic OS=Homo sapiens OX=9606 GN=IDH1 PE=1 SV=2	29
8	61,630	sp|P00558|PGK1_HUMAN	Phosphoglycerate kinase 1 OS=Homo sapiens OX=9606 GN=PGK1 PE=1 SV=3	22
7	9,091	sp|P15924|DESP_HUMAN	Desmoplakin OS=Homo sapiens OX=9606 GN=DSP PE=1 SV=3	20
12	51,050	sp|Q9BXN1|ASPN_HUMAN	Asporin OS=Homo sapiens OX=9606 GN=ASPN PE=1 SV=2	18
13	55,850	sp|P16930|FAAA_HUMAN	Fumarylacetoacetase OS=Homo sapiens OX=9606 GN=FAH PE=1 SV=2	17
16	33,500	sp|P00966|ASSY_HUMAN	Argininosuccinate synthase OS=Homo sapiens OX=9606 GN=ASS1 PE=1 SV=2	13
15	18,120	sp|P14923|PLAK_HUMAN	Junction plakoglobin OS=Homo sapiens OX=9606 GN=JUP PE=1 SV=3	11
17	76,870	sp|P68871|HBB_HUMAN	Hemoglobin subunit beta OS=Homo sapiens OX=9606 GN=HBB PE=1 SV=2	10
18	34,700	sp|Q92947|GCDH_HUMAN	Glutaryl-CoA dehydrogenase, mitochondrial OS=Homo sapiens OX=9606 GN=GCDH PE=1 SV=1	10
27	35,160	sp|P04075|ALDOA_HUMAN	Fructose-bisphosphate aldolase A OS=Homo sapiens OX=9606 GN=ALDOA PE=1 SV=2	9

21	13,160	sp|Q02413|DSG1_HUMAN	Desmoglein-1 OS=Homo sapiens OX=9606 GN=DSG1 PE=1 SV=2	8
23	24,410	sp|P06732|KCRM_HUMAN	Creatine kinase M-type OS=Homo sapiens OX=9606 GN=CKM PE=1 SV=2	8
32	15,970	sp|P51888|PRELP_HUMAN	Prolargin OS=Homo sapiens OX=9606 GN=PRELP PE=1 SV=1	8
19	23,600	sp|P52209|6PGD_HUMAN	6-phosphogluconate dehydrogenase, decarboxylating OS=Homo sapiens OX=9606 GN=PGD PE=1 SV=3	7
20	28,060	sp|P0DOX5|IGG1_HUMAN	Immunoglobulin gamma-1 heavy chain OS=Homo sapiens OX=9606 PE=1 SV=2	7
22	15,260	sp|Q86YZ3|HORN_HUMAN	Hornerin OS=Homo sapiens OX=9606 GN=HRNR PE=1 SV=2	7
24	23,180	sp|O75390|CISY_HUMAN	Citrate synthase, mitochondrial OS=Homo sapiens OX=9606 GN=CS PE=1 SV=2	7
25	15,980	sp|P06396|GELS_HUMAN	Gelsolin OS=Homo sapiens OX=9606 GN=GSN PE=1 SV=1	7
26	21,410	sp|P22695|QCR2_HUMAN	Cytochrome b-c1 complex subunit 2, mitochondrial OS=Homo sapiens OX=9606 GN=UQCRC2 PE=1 SV=3	7
29	15,370	sp|Q5VTE0|EF1A3_HUMAN	Putative elongation factor 1-alpha-like 3 OS=Homo sapiens OX=9606 GN=EEF1A1P5 PE=5 SV=1	7
30	10,280	sp|P02671|FIBA_HUMAN	Fibrinogen alpha chain OS=Homo sapiens OX=9606 GN=FGA PE=1 SV=2	7
33	26,060	sp|P26440|IVD_HUMAN	Isovaleryl-CoA dehydrogenase, mitochondrial OS=Homo sapiens OX=9606 GN=IVD PE=1 SV=2	7
28	7,714	sp|O00159|MYO1C_HUMAN	Unconventional myosin-Ic OS=Homo sapiens OX=9606 GN=MYO1C PE=1 SV=4	6
31	25,450	sp|Q99536|VAT1_HUMAN	Synaptic vesicle membrane protein VAT-1 homolog OS=Homo sapiens OX=9606 GN=VAT1 PE=1 SV=2	6
36	31,800	sp|P00915|CAH1_HUMAN	Carbonic anhydrase 1 OS=Homo sapiens OX=9606 GN=CA1 PE=1 SV=2	6
42	48,590	sp|P69905|HBA_HUMAN	Hemoglobin subunit alpha OS=Homo sapiens OX=9606 GN=HBA1 PE=1 SV=2	6
35	15,760	sp|P22234|PUR6_HUMAN	Multifunctional protein ADE2 OS=Homo sapiens OX=9606 GN=PAICS PE=1 SV=3	5
37	13,720	sp|P49411|EFTU_HUMAN	Elongation factor Tu, mitochondrial OS=Homo sapiens OX=9606 GN=TUFM PE=1 SV=2	4
38	16,530	sp|P30533|AMRP_HUMAN	Alpha-2-macroglobulin receptor-associated protein OS=Homo sapiens OX=9606 GN=LRPAP1 PE=1 SV=1	4
39	8,974	sp|Q99798|ACON_HUMAN	Aconitate hydratase, mitochondrial OS=Homo sapiens OX=9606 GN=ACO2 PE=1 SV=2	4
40	17,630	sp|P42765|THIM_HUMAN	3-ketoacyl-CoA thiolase, mitochondrial OS=Homo sapiens OX=9606 GN=ACAA2 PE=1 SV=2	4

41	4,726	sp|Q5D862|FILA2_HUMAN	Filaggrin-2 OS=Homo sapiens OX=9606 GN=FLG2 PE=1 SV=1	4
43	11,640	sp|P11310|ACADM_HUMAN	Medium-chain specific acyl-CoA dehydrogenase, mitochondrial OS=Homo sapiens OX=9606 GN=ACADM PE=1 SV=1	4
44	4,089	sp|P42704|LPPRC_HUMAN	Leucine-rich PPR motif-containing protein, mitochondrial OS=Homo sapiens OX=9606 GN=LRPPRC PE=1 SV=3	4
45	12,380	sp|Q96IJ6|GMPPA_HUMAN	Mannose-1-phosphate guanyltransferase alpha OS=Homo sapiens OX=9606 GN=GMPPA PE=1 SV=1	4
52	14,360	sp|P61163|ACTZ_HUMAN	Alpha-centractin OS=Homo sapiens OX=9606 GN=ACTR1A PE=1 SV=1	4
64	12,150	sp|P07477|TRY1_HUMAN	Trypsin-1 OS=Homo sapiens OX=9606 GN=PRSS1 PE=1 SV=1	4
46	4,362	sp|Q08554|DSC1_HUMAN	Desmocollin-1 OS=Homo sapiens OX=9606 GN=DSC1 PE=1 SV=2	3
48	12,800	sp|P63261|ACTG_HUMAN	Actin, cytoplasmic 2 OS=Homo sapiens OX=9606 GN=ACTG1 PE=1 SV=1	3
49	12,880	sp|P01859|IGHG2_HUMAN	Immunoglobulin heavy constant gamma 2 OS=Homo sapiens OX=9606 GN=IGHG2 PE=1 SV=2	3
50	12,440	sp|Q8IWB7|WDFY1_HUMAN	WD repeat and FYVE domain-containing protein 1 OS=Homo sapiens OX=9606 GN=WDFY1 PE=1 SV=1	3
53	9,014	sp|Q14103|HNRPD_HUMAN	Heterogeneous nuclear ribonucleoprotein D0 OS=Homo sapiens OX=9606 GN=HNRNPD PE=1 SV=1	3
54	4,949	sp|Q9UHD8|SEPT9_HUMAN	Septin-9 OS=Homo sapiens OX=9606 GN=SEPTIN9 PE=1 SV=2	3
58	7,343	sp|Q9H9P8|L2HDH_HUMAN	L-2-hydroxyglutarate dehydrogenase, mitochondrial OS=Homo sapiens OX=9606 GN=L2HGDH PE=1 SV=3	3
63	5,541	sp|Q12797|ASPH_HUMAN	Aspartyl/asparaginyl beta-hydroxylase OS=Homo sapiens OX=9606 GN=ASPH PE=1 SV=3	3
51	9,851	sp|P04406|G3P_HUMAN	Glyceraldehyde-3-phosphate dehydrogenase OS=Homo sapiens OX=9606 GN=GAPDH PE=1 SV=3	2
55	3,627	sp|Q5T749|KPRP_HUMAN	Keratinocyte proline-rich protein OS=Homo sapiens OX=9606 GN=KPRP PE=1 SV=1	2
56	7,111	sp|Q9NS86|LANC2_HUMAN	LanC-like protein 2 OS=Homo sapiens OX=9606 GN=LANCL2 PE=1 SV=1	2
57	9,145	sp|P07355|ANXA2_HUMAN	Annexin A2 OS=Homo sapiens OX=9606 GN=ANXA2 PE=1 SV=2	2
59	7,949	sp|Q6NZI2|CAVN1_HUMAN	Caveolae-associated protein 1 OS=Homo sapiens OX=9606 GN=CAVIN1 PE=1 SV=1	2

60	6,964	sp|P07585|PGS2_HUMAN	Decorin OS=Homo sapiens OX=9606 GN=DCN PE=1 SV=1	2
61	3,009	sp|P33121|ACSL1_HUMAN	Long-chain-fatty-acid--CoA ligase 1 OS=Homo sapiens OX=9606 GN=ACSL1 PE=1 SV=1	2
62	5,556	sp|P00367|DHE3_HUMAN	Glutamate dehydrogenase 1, mitochondrial OS=Homo sapiens OX=9606 GN=GLUD1 PE=1 SV=2	2
67	1,639	sp|Q96C24|SYTL4_HUMAN	Synaptotagmin-like protein 4 OS=Homo sapiens OX=9606 GN=SYTL4 PE=1 SV=2	2
71	15,750	sp|Q9NZT1|CALL5_HUMAN	Calmodulin-like protein 5 OS=Homo sapiens OX=9606 GN=CALML5 PE=1 SV=2	2
76	3,826	sp|P10636|TAU_HUMAN	Microtubule-associated protein tau OS=Homo sapiens OX=9606 GN=MAPT PE=1 SV=5	2
**Non-COVID_Spot 3 (Proteins)**				
2	76,960	sp|Q86TX2|ACOT1_HUMAN	Acyl-coenzyme A thioesterase 1 OS=Homo sapiens OX=9606 GN=ACOT1 PE=1 SV=1	33
53	68,320	sp|P49753|ACOT2_HUMAN	Acyl-coenzyme A thioesterase 2, mitochondrial OS=Homo sapiens OX=9606 GN=ACOT2 PE=1 SV=6	33
7	61,210	sp|P42765|THIM_HUMAN	3-ketoacyl-CoA thiolase, mitochondrial OS=Homo sapiens OX=9606 GN=ACAA2 PE=1 SV=2	22
6	56,840	sp|Q9BXN1|ASPN_HUMAN	Asporin OS=Homo sapiens OX=9606 GN=ASPN PE=1 SV=2	20
8	38,410	sp|O75390|CISY_HUMAN	Citrate synthase, mitochondrial OS=Homo sapiens OX=9606 GN=CS PE=1 SV=2	17
11	20,210	sp|P02671|FIBA_HUMAN	Fibrinogen alpha chain OS=Homo sapiens OX=9606 GN=FGA PE=1 SV=2	17
9	49,750	sp|P62195|PRS8_HUMAN	26S proteasome regulatory subunit 8 OS=Homo sapiens OX=9606 GN=PSMC5 PE=1 SV=1	16
10	48,440	sp|P00558|PGK1_HUMAN	Phosphoglycerate kinase 1 OS=Homo sapiens OX=9606 GN=PGK1 PE=1 SV=3	15
12	47,580	sp|Q99536|VAT1_HUMAN	Synaptic vesicle membrane protein VAT-1 homolog OS=Homo sapiens OX=9606 GN=VAT1 PE=1 SV=2	14
15	21,870	sp|P06396|GELS_HUMAN	Gelsolin OS=Homo sapiens OX=9606 GN=GSN PE=1 SV=1	14
13	29,710	sp|O75874|IDHC_HUMAN	Isocitrate dehydrogenase [NADP] cytoplasmic OS=Homo sapiens OX=9606 GN=IDH1 PE=1 SV=2	11
14	34,220	sp|P00966|ASSY_HUMAN	Argininosuccinate synthase OS=Homo sapiens OX=9606 GN=ASS1 PE=1 SV=2	11
22	14,640	sp|Q12797|ASPH_HUMAN	Aspartyl/asparaginyl beta-hydroxylase OS=Homo sapiens OX=9606 GN=ASPH PE=1 SV=3	10
16	76,870	sp|P68871|HBB_HUMAN	Hemoglobin subunit beta OS=Homo sapiens OX=9606 GN=HBB PE=1 SV=2	9
18	6,615	sp|P01024|CO3_HUMAN	Complement C3 OS=Homo sapiens OX=9606 GN=C3 PE=1 SV=2	9

19	32,650	sp|Q92947|GCDH_HUMAN	Glutaryl-CoA dehydrogenase, mitochondrial OS=Homo sapiens OX=9606 GN=GCDH PE=1 SV=1	9
17	22,470	sp|Q9NTK5|OLA1_HUMAN	Obg-like ATPase 1 OS=Homo sapiens OX=9606 GN=OLA1 PE=1 SV=2	8
21	29,840	sp|P0DOX5|IGG1_HUMAN	Immunoglobulin gamma-1 heavy chain OS=Homo sapiens OX=9606 PE=1 SV=2	8
24	21,220	sp|P09622|DLDH_HUMAN	Dihydrolipoyl dehydrogenase, mitochondrial OS=Homo sapiens OX=9606 GN=DLD PE=1 SV=2	8
58	12,150	sp|P07477|TRY1_HUMAN	Trypsin-1 OS=Homo sapiens OX=9606 GN=PRSS1 PE=1 SV=1	8
20	21,950	sp|Q8IWB7|WDFY1_HUMAN	WD repeat and FYVE domain-containing protein 1 OS=Homo sapiens OX=9606 GN=WDFY1 PE=1 SV=1	7
23	25,260	sp|P52209|6PGD_HUMAN	6-phosphogluconate dehydrogenase, decarboxylating OS=Homo sapiens OX=9606 GN=PGD PE=1 SV=3	7
28	10,900	sp|Q99798|ACON_HUMAN	Aconitate hydratase, mitochondrial OS=Homo sapiens OX=9606 GN=ACO2 PE=1 SV=2	6
30	52,110	sp|P69905|HBA_HUMAN	Hemoglobin subunit alpha OS=Homo sapiens OX=9606 GN=HBA1 PE=1 SV=2	6
25	20,610	sp|P07585|PGS2_HUMAN	Decorin OS=Homo sapiens OX=9606 GN=DCN PE=1 SV=1	5
26	19,860	sp|Q6YN16|HSDL2_HUMAN	Hydroxysteroid dehydrogenase-like protein 2 OS=Homo sapiens OX=9606 GN=HSDL2 PE=1 SV=1	5
27	22,310	sp|P06732|KCRM_HUMAN	**Creatine kinase M-type** OS=Homo sapiens OX=9606 GN=CKM PE=1 SV=2	5
31	15,930	sp|P48735|IDHP_HUMAN	Isocitrate dehydrogenase [NADP], mitochondrial OS=Homo sapiens OX=9606 GN=IDH2 PE=1 SV=2	5
36	20,330	sp|P04075|ALDOA_HUMAN	Fructose-bisphosphate aldolase A OS=Homo sapiens OX=9606 GN=ALDOA PE=1 SV=2	5
51	21,470	sp|P01859|IGHG2_HUMAN	Immunoglobulin heavy constant gamma 2 OS=Homo sapiens OX=9606 GN=IGHG2 PE=1 SV=2	5
32	14,510	sp|P07954|FUMH_HUMAN	Fumarate hydratase, mitochondrial OS=Homo sapiens OX=9606 GN=FH PE=1 SV=3	4
34	16,530	sp|P30533|AMRP_HUMAN	Alpha-2-macroglobulin receptor-associated protein OS=Homo sapiens OX=9606 GN=LRPAP1 PE=1 SV=1	4
35	13,690	sp|P22695|QCR2_HUMAN	Cytochrome b-c1 complex subunit 2, mitochondrial OS=Homo sapiens OX=9606 GN=UQCRC2 PE=1 SV=3	4
37	13,870	sp|P51888|PRELP_HUMAN	Prolargin OS=Homo sapiens OX=9606 GN=PRELP PE=1 SV=1	4

38	14,510	sp|Q86VN1|VPS36_HUMAN	Vacuolar protein-sorting-associated protein 36 OS=Homo sapiens OX=9606 GN=VPS36 PE=1 SV=1	4
39	12,960	sp|Q14103|HNRPD_HUMAN	Heterogeneous nuclear ribonucleoprotein D0 OS=Homo sapiens OX=9606 GN=HNRNPD PE=1 SV=1	4
40	7,163	sp|P33121|ACSL1_HUMAN	Long-chain-fatty-acid--CoA ligase 1 OS=Homo sapiens OX=9606 GN=ACSL1 PE=1 SV=1	4
46	17,270	sp|Q5VW32|BROX_HUMAN	BRO1 domain-containing protein BROX OS=Homo sapiens OX=9606 GN=BROX PE=1 SV=1	4
41	8,571	sp|Q96IJ6|GMPPA_HUMAN	Mannose-1-phosphate guanyltransferase alpha OS=Homo sapiens OX=9606 GN=GMPPA PE=1 SV=1	3
42	11,230	sp|P11766|ADHX_HUMAN	Alcohol dehydrogenase class-3 OS=Homo sapiens OX=9606 GN=ADH5 PE=1 SV=4	3
47	6,989	sp|P00367|DHE3_HUMAN	Glutamate dehydrogenase 1, mitochondrial OS=Homo sapiens OX=9606 GN=GLUD1 PE=1 SV=2	2
43	5,235	sp|P14923|PLAK_HUMAN	Junction plakoglobin OS=Homo sapiens OX=9606 GN=JUP PE=1 SV=3	3
49	0,560	sp|Q09666|AHNK_HUMAN	Neuroblast differentiation-associated protein AHNAK OS=Homo sapiens OX=9606 GN=AHNAK PE=1 SV=2	2
44	11,040	sp|Q5VTE0|EF1A3_HUMAN	Putative elongation factor 1-alpha-like 3 OS=Homo sapiens OX=9606 GN=EEF1A1P5 PE=5 SV=1	3
45	9,091	sp|P50454|SERPH_HUMAN	Serpin H1 OS=Homo sapiens OX=9606 GN=SERPINH1 PE=1 SV=2	3
52	7,733	sp|P63261|ACTG_HUMAN	Actin, cytoplasmic 2 OS=Homo sapiens OX=9606 GN=ACTG1 PE=1 SV=1	2
48	48,600	sp|P01834|IGKC_HUMAN	Immunoglobulin kappa constant OS=Homo sapiens OX=9606 GN=IGKC PE=1 SV=2	3
54	4,394	sp|O15523|DDX3Y_HUMAN	ATP-dependent RNA helicase DDX3Y OS=Homo sapiens OX=9606 GN=DDX3Y PE=1 SV=2	2
55	5,251	sp|P16930|FAAA_HUMAN	Fumarylacetoacetase OS=Homo sapiens OX=9606 GN=FAH PE=1 SV=2	2
56	6,651	sp|P11310|ACADM_HUMAN	Medium-chain specific acyl-CoA dehydrogenase, mitochondrial OS=Homo sapiens OX=9606 GN=ACADM PE=1 SV=1	2
57	4,930	sp|P26440|IVD_HUMAN	Isovaleryl-CoA dehydrogenase, mitochondrial OS=Homo sapiens OX=9606 GN=IVD PE=1 SV=2	2
50	1,567	sp|P15924|DESP_HUMAN	Desmoplakin OS=Homo sapiens OX=9606 GN=DSP PE=1 SV=3	3

59	8,967	sp|P21810|PGS1_HUMAN	Biglycan OS=Homo sapiens OX=9606 GN=BGN PE=1 SV=2	3
60	3,244	sp|Q08554|DSC1_HUMAN	Desmocollin-1 OS=Homo sapiens OX=9606 GN=DSC1 PE=1 SV=2	2
**Post-COVID_Spot 1 (Proteins)**				
1	81,100	sp|P06732|KCRM_HUMAN	**Creatine kinase M-type** OS=Homo sapiens OX=9606 GN=CKM PE=1 SV=2	71
11	37,420	sp|P01859|IGHG2_HUMAN	Immunoglobulin heavy constant gamma 2 OS=Homo sapiens OX=9606 GN=IGHG2 PE=1 SV=2	16
6	50,670	sp|P60709|ACTB_HUMAN	Actin, cytoplasmic 1 OS=Homo sapiens OX=9606 GN=ACTB PE=1 SV=1	13
7	36,320	sp|Q9BXN1|ASPN_HUMAN	Asporin OS=Homo sapiens OX=9606 GN=ASPN PE=1 SV=2	13
15	40,580	sp|P62736|ACTA_HUMAN	Actin, aortic smooth muscle OS=Homo sapiens OX=9606 GN=ACTA2 PE=1 SV=1	13
8	9,020	sp|P01024|CO3_HUMAN	Complement C3 OS=Homo sapiens OX=9606 GN=C3 PE=1 SV=2	12
9	32,300	sp|P49411|EFTU_HUMAN	Elongation factor Tu, mitochondrial OS=Homo sapiens OX=9606 GN=TUFM PE=1 SV=2	11
10	35,560	sp|P16930|FAAA_HUMAN	Fumarylacetoacetase OS=Homo sapiens OX=9606 GN=FAH PE=1 SV=2	10
36	12,150	sp|P07477|TRY1_HUMAN	Trypsin-1 OS=Homo sapiens OX=9606 GN=PRSS1 PE=1 SV=1	10
12	63,950	sp|P68871|HBB_HUMAN	Hemoglobin subunit beta OS=Homo sapiens OX=9606 GN=HBB PE=1 SV=2	8
13	17,800	sp|P51888|PRELP_HUMAN	Prolargin OS=Homo sapiens OX=9606 GN=PRELP PE=1 SV=1	6
14	18,720	sp|P00738|HPT_HUMAN	Haptoglobin OS=Homo sapiens OX=9606 GN=HP PE=1 SV=1	6
16	19,320	sp|O75874|IDHC_HUMAN	Isocitrate dehydrogenase [NADP] cytoplasmic OS=Homo sapiens OX=9606 GN=IDH1 PE=1 SV=2	6
24	19,150	sp|P0DOX5|IGG1_HUMAN	Immunoglobulin gamma-1 heavy chain OS=Homo sapiens OX=9606 PE=1 SV=2	6
17	17,020	sp|P61163|ACTZ_HUMAN	Alpha-centractin OS=Homo sapiens OX=9606 GN=ACTR1A PE=1 SV=1	5
23	17,880	sp|Q86VN1|VPS36_HUMAN	Vacuolar protein-sorting-associated protein 36 OS=Homo sapiens OX=9606 GN=VPS36 PE=1 SV=1	5
19	6,120	sp|P02671|FIBA_HUMAN	Fibrinogen alpha chain OS=Homo sapiens OX=9606 GN=FGA PE=1 SV=2	4
20	9,079	sp|P06396|GELS_HUMAN	Gelsolin OS=Homo sapiens OX=9606 GN=GSN PE=1 SV=1	4
21	4,519	sp|P42704|LPPRC_HUMAN	Leucine-rich PPR motif-containing protein, mitochondrial OS=Homo sapiens OX=9606 GN=LRPPRC PE=1 SV=3	4

25	14,780	sp|P62195|PRS8_HUMAN	26S proteasome regulatory subunit 8 OS=Homo sapiens OX=9606 GN=PSMC5 PE=1 SV=1	4
28	10,550	sp|P00558|PGK1_HUMAN	Phosphoglycerate kinase 1 OS=Homo sapiens OX=9606 GN=PGK1 PE=1 SV=3	4
30	10,770	sp|P08559|ODPA_HUMAN	Pyruvate dehydrogenase E1 component subunit alpha, somatic form, mitochondrial OS=Homo sapiens OX=9606 GN=PDHA1 PE=1 SV=3	4
31	15,930	sp|P04075|ALDOA_HUMAN	Fructose-bisphosphate aldolase A OS=Homo sapiens OX=9606 GN=ALDOA PE=1 SV=2	4
22	9,938	sp|P52209|6PGD_HUMAN	6-phosphogluconate dehydrogenase, decarboxylating OS=Homo sapiens OX=9606 GN=PGD PE=1 SV=3	3
27	10,140	sp|Q14103|HNRPD_HUMAN	Heterogeneous nuclear ribonucleoprotein D0 OS=Homo sapiens OX=9606 GN=HNRNPD PE=1 SV=1	3
29	8,102	sp|P23526|SAHH_HUMAN	Adenosylhomocysteinase OS=Homo sapiens OX=9606 GN=AHCY PE=1 SV=4	3
32	11,400	sp|Q86TX2|ACOT1_HUMAN	Acyl-coenzyme A thioesterase 1 OS=Homo sapiens OX=9606 GN=ACOT1 PE=1 SV=1	3
38	16,900	sp|P69905|HBA_HUMAN	Hemoglobin subunit alpha OS=Homo sapiens OX=9606 GN=HBA1 PE=1 SV=2	3
33	3,464	sp|P02545|LMNA_HUMAN	Prelamin-A/C OS=Homo sapiens OX=9606 GN=LMNA PE=1 SV=1	2
34	6,684	sp|Q99447|PCY2_HUMAN	Ethanolamine-phosphate cytidylyltransferase OS=Homo sapiens OX=9606 GN=PCYT2 PE=1 SV=1	2
35	4,978	sp|Q5VTE0|EF1A3_HUMAN	Putative elongation factor 1-alpha-like 3 OS=Homo sapiens OX=9606 GN=EEF1A1P5 PE=5 SV=1	2
39	5,660	sp|Q6ZMU5|TRI72_HUMAN	Tripartite motif-containing protein 72 OS=Homo sapiens OX=9606 GN=TRIM72 PE=1 SV=2	2
41	7,221	sp|Q9Y697|NFS1_HUMAN	Cysteine desulfurase, mitochondrial OS=Homo sapiens OX=9606 GN=NFS1 PE=1 SV=3	2
42	10,340	sp|P00915|CAH1_HUMAN	Carbonic anhydrase 1 OS=Homo sapiens OX=9606 GN=CA1 PE=1 SV=2	2
44	1,698	sp|P11498|PYC_HUMAN	Pyruvate carboxylase, mitochondrial OS=Homo sapiens OX=9606 GN=PC PE=1 SV=2	2
53	8,023	sp|Q8WTS1|ABHD5_HUMAN	1-acylglycerol-3-phosphate O-acyltransferase ABHD5 OS=Homo sapiens OX=9606 GN=ABHD5 PE=1 SV=1	2
58	3,766	sp|P14618|KPYM_HUMAN	Pyruvate kinase PKM OS=Homo sapiens OX=9606 GN=PKM PE=1 SV=4	2
64	3,826	sp|P10636|TAU_HUMAN	Microtubule-associated protein tau OS=Homo sapiens OX=9606 GN=MAPT PE=1 SV=5	2
**Post-COVID_Spot 2 (Proteins)**				

3	78,740	sp|P06732|KCRM_HUMAN	**Creatine kinase M-type** OS=Homo sapiens OX=9606 GN=CKM PE=1 SV=2	110
5	42,450	sp|P00558|PGK1_HUMAN	Phosphoglycerate kinase 1 OS=Homo sapiens OX=9606 GN=PGK1 PE=1 SV=3	14
8	42,000	sp|P17540|KCRS_HUMAN	**Creatine kinase S-type, mitochondrial** OS=Homo sapiens OX=9606 GN=CKMT2 PE=1 SV=2	14
6	39,860	sp|O75874|IDHC_HUMAN	Isocitrate dehydrogenase [NADP] cytoplasmic OS=Homo sapiens OX=9606 GN=IDH1 PE=1 SV=2	13
10	36,580	sp|Q9BXN1|ASPN_HUMAN	Asporin OS=Homo sapiens OX=9606 GN=ASPN PE=1 SV=2	13
9	44,530	sp|P60709|ACTB_HUMAN	Actin, cytoplasmic 1 OS=Homo sapiens OX=9606 GN=ACTB PE=1 SV=1	12
11	42,720	sp|P16930|FAAA_HUMAN	Fumarylacetoacetase OS=Homo sapiens OX=9606 GN=FAH PE=1 SV=2	11
17	36,500	sp|P01859|IGHG2_HUMAN	Immunoglobulin heavy constant gamma 2 OS=Homo sapiens OX=9606 GN=IGHG2 PE=1 SV=2	11
12	65,990	sp|P68871|HBB_HUMAN	Hemoglobin subunit beta OS=Homo sapiens OX=9606 GN=HBB PE=1 SV=2	8
13	27,390	sp|P0DOX5|IGG1_HUMAN	Immunoglobulin gamma-1 heavy chain OS=Homo sapiens OX=9606 PE=1 SV=2	8
18	20,330	sp|P04075|ALDOA_HUMAN	Fructose-bisphosphate aldolase A OS=Homo sapiens OX=9606 GN=ALDOA PE=1 SV=2	8
23	32,630	sp|P68133|ACTS_HUMAN	Actin, alpha skeletal muscle OS=Homo sapiens OX=9606 GN=ACTA1 PE=1 SV=1	8
35	13,770	sp|P07477|TRY1_HUMAN	Trypsin-1 OS=Homo sapiens OX=9606 GN=PRSS1 PE=1 SV=1	8
20	24,230	sp|P07451|CAH3_HUMAN	Carbonic anhydrase 3 OS=Homo sapiens OX=9606 GN=CA3 PE=1 SV=3	7
16	18,720	sp|P00738|HPT_HUMAN	Haptoglobin OS=Homo sapiens OX=9606 GN=HP PE=1 SV=1	6
15	15,910	sp|Q86TX2|ACOT1_HUMAN	Acyl-coenzyme A thioesterase 1 OS=Homo sapiens OX=9606 GN=ACOT1 PE=1 SV=1	5
19	16,590	sp|P49411|EFTU_HUMAN	Elongation factor Tu, mitochondrial OS=Homo sapiens OX=9606 GN=TUFM PE=1 SV=2	5
21	6,928	sp|P02671|FIBA_HUMAN	Fibrinogen alpha chain OS=Homo sapiens OX=9606 GN=FGA PE=1 SV=2	5
22	10,560	sp|P52209|6PGD_HUMAN	6-phosphogluconate dehydrogenase, decarboxylating OS=Homo sapiens OX=9606 GN=PGD PE=1 SV=3	4
25	8,824	sp|P06396|GELS_HUMAN	Gelsolin OS=Homo sapiens OX=9606 GN=GSN PE=1 SV=1	4
28	12,390	sp|Q14103|HNRPD_HUMAN	Heterogeneous nuclear ribonucleoprotein D0 OS=Homo sapiens OX=9606 GN=HNRNPD PE=1 SV=1	4
29	3,848	sp|P01024|CO3_HUMAN	Complement C3 OS=Homo sapiens OX=9606 GN=C3 PE=1 SV=2	4
30	28,170	sp|P69905|HBA_HUMAN	Hemoglobin subunit alpha OS=Homo sapiens OX=9606 GN=HBA1 PE=1 SV=2	4

26	8,102	sp|P23526|SAHH_HUMAN	Adenosylhomocysteinase OS=Homo sapiens OX=9606 GN=AHCY PE=1 SV=4	3
27	10,470	sp|P51888|PRELP_HUMAN	Prolargin OS=Homo sapiens OX=9606 GN=PRELP PE=1 SV=1	3
33	8,175	sp|Q86YZ3|HORN_HUMAN	Hornerin OS=Homo sapiens OX=9606 GN=HRNR PE=1 SV=2	3
31	8,312	sp|P42765|THIM_HUMAN	3-ketoacyl-CoA thiolase, mitochondrial OS=Homo sapiens OX=9606 GN=ACAA2 PE=1 SV=2	2
32	3,590	sp|Q99798|ACON_HUMAN	Aconitate hydratase, mitochondrial OS=Homo sapiens OX=9606 GN=ACO2 PE=1 SV=2	2
34	7,576	sp|Q5VTE0|EF1A3_HUMAN	Putative elongation factor 1-alpha-like 3 OS=Homo sapiens OX=9606 GN=EEF1A1P5 PE=5 SV=1	2
36	7,990	sp|P17174|AATC_HUMAN	Aspartate aminotransferase, cytoplasmic OS=Homo sapiens OX=9606 GN=GOT1 PE=1 SV=3	2
37	7,775	sp|P15104|GLNA_HUMAN	Glutamine synthetase OS=Homo sapiens OX=9606 GN=GLUL PE=1 SV=4	2
38	0,944	sp|P12111|CO6A3_HUMAN	Collagen alpha-3(VI) chain OS=Homo sapiens OX=9606 GN=COL6A3 PE=1 SV=5	2
47	5,263	sp|P36955|PEDF_HUMAN	Pigment epithelium-derived factor OS=Homo sapiens OX=9606 GN=SERPINF1 PE=1 SV=4	2
**Post-COVID_Spot 3 (Proteins)**				
4	84,780	sp|P06732|KCRM_HUMAN	**Creatine kinase M-type** OS=Homo sapiens OX=9606 GN=CKM PE=1 SV=2	139
2	19,850	sp|P15924|DESP_HUMAN	Desmoplakin OS=Homo sapiens OX=9606 GN=DSP PE=1 SV=3	43
12	19,830	sp|Q02413|DSG1_HUMAN	Desmoglein-1 OS=Homo sapiens OX=9606 GN=DSG1 PE=1 SV=2	15
9	22,280	sp|P14923|PLAK_HUMAN	Junction plakoglobin OS=Homo sapiens OX=9606 GN=JUP PE=1 SV=3	14
13	38,400	sp|P60709|ACTB_HUMAN	Actin, cytoplasmic 1 OS=Homo sapiens OX=9606 GN=ACTB PE=1 SV=1	11
14	32,630	sp|Q9BXN1|ASPN_HUMAN	Asporin OS=Homo sapiens OX=9606 GN=ASPN PE=1 SV=2	11
18	43,330	sp|P01857|IGHG1_HUMAN	Immunoglobulin heavy constant gamma 1 OS=Homo sapiens OX=9606 GN=IGHG1 PE=1 SV=1	11
15	42,960	sp|P17540|KCRS_HUMAN	**Creatine kinase S-type, mitochondrial** OS=Homo sapiens OX=9606 GN=CKMT2 PE=1 SV=2	10
20	36,500	sp|P01859|IGHG2_HUMAN	Immunoglobulin heavy constant gamma 2 OS=Homo sapiens OX=9606 GN=IGHG2 PE=1 SV=2	10
16	6,735	sp|P01024|CO3_HUMAN	Complement C3 OS=Homo sapiens OX=9606 GN=C3 PE=1 SV=2	9

19	30,400	sp|Q86TX2|ACOT1_HUMAN	Acyl-coenzyme A thioesterase 1 OS=Homo sapiens OX=9606 GN=ACOT1 PE=1 SV=1	9
31	24,670	sp|P68133|ACTS_HUMAN	Actin, alpha skeletal muscle OS=Homo sapiens OX=9606 GN=ACTA1 PE=1 SV=1	9
21	31,320	sp|P04075|ALDOA_HUMAN	Fructose-bisphosphate aldolase A OS=Homo sapiens OX=9606 GN=ALDOA PE=1 SV=2	8
45	12,150	sp|P07477|TRY1_HUMAN	Trypsin-1 OS=Homo sapiens OX=9606 GN=PRSS1 PE=1 SV=1	8
25	26,870	sp|P04406|G3P_HUMAN	Glyceraldehyde-3-phosphate dehydrogenase OS=Homo sapiens OX=9606 GN=GAPDH PE=1 SV=3	6
32	10,670	sp|Q86YZ3|HORN_HUMAN	Hornerin OS=Homo sapiens OX=9606 GN=HRNR PE=1 SV=2	6
23	17,510	sp|P00558|PGK1_HUMAN	Phosphoglycerate kinase 1 OS=Homo sapiens OX=9606 GN=PGK1 PE=1 SV=3	5
24	45,580	sp|P68871|HBB_HUMAN	Hemoglobin subunit beta OS=Homo sapiens OX=9606 GN=HBB PE=1 SV=2	5
26	13,130	sp|Q9NTK5|OLA1_HUMAN	Obg-like ATPase 1 OS=Homo sapiens OX=9606 GN=OLA1 PE=1 SV=2	5
27	11,840	sp|O75874|IDHC_HUMAN	Isocitrate dehydrogenase [NADP] cytoplasmic OS=Homo sapiens OX=9606 GN=IDH1 PE=1 SV=2	4
28	40,440	sp|P47929|LEG7_HUMAN	Galectin-7 OS=Homo sapiens OX=9606 GN=LGALS7 PE=1 SV=2	4
36	28,170	sp|P69905|HBA_HUMAN	Hemoglobin subunit alpha OS=Homo sapiens OX=9606 GN=HBA1 PE=1 SV=2	4
50	41,100	sp|Q9NZT1|CALL5_HUMAN	Calmodulin-like protein 5 OS=Homo sapiens OX=9606 GN=CALML5 PE=1 SV=2	4
29	9,785	sp|P16930|FAAA_HUMAN	Fumarylacetoacetase OS=Homo sapiens OX=9606 GN=FAH PE=1 SV=2	3
30	11,450	sp|Q99536|VAT1_HUMAN	Synaptic vesicle membrane protein VAT-1 homolog OS=Homo sapiens OX=9606 GN=VAT1 PE=1 SV=2	3
33	9,981	sp|P14618|KPYM_HUMAN	Pyruvate kinase PKM OS=Homo sapiens OX=9606 GN=PKM PE=1 SV=4	3
34	11,920	sp|P07451|CAH3_HUMAN	Carbonic anhydrase 3 OS=Homo sapiens OX=9606 GN=CA3 PE=1 SV=3	3
35	4,362	sp|Q08554|DSC1_HUMAN	Desmocollin-1 OS=Homo sapiens OX=9606 GN=DSC1 PE=1 SV=2	3
38	10,100	sp|P00738|HPT_HUMAN	Haptoglobin OS=Homo sapiens OX=9606 GN=HP PE=1 SV=1	3
39	9,014	sp|Q14103|HNRPD_HUMAN	Heterogeneous nuclear ribonucleoprotein D0 OS=Homo sapiens OX=9606 GN=HNRNPD PE=1 SV=1	3
40	12,060	sp|P15104|GLNA_HUMAN	Glutamine synthetase OS=Homo sapiens OX=9606 GN=GLUL PE=1 SV=4	3
41	3,325	sp|P06396|GELS_HUMAN	Gelsolin OS=Homo sapiens OX=9606 GN=GSN PE=1 SV=1	2
42	7,965	sp|P49411|EFTU_HUMAN	Elongation factor Tu, mitochondrial OS=Homo sapiens OX=9606 GN=TUFM PE=1 SV=2	2

43	8,123	sp|P30533|AMRP_HUMAN	Alpha-2-macroglobulin receptor-associated protein OS=Homo sapiens OX=9606 GN=LRPAP1 PE=1 SV=1	2
44	7,940	sp|Q15517|CDSN_HUMAN	Corneodesmosin OS=Homo sapiens OX=9606 GN=CDSN PE=1 SV=3	2
46	5,385	sp|Q99798|ACON_HUMAN	Aconitate hydratase, mitochondrial OS=Homo sapiens OX=9606 GN=ACO2 PE=1 SV=2	2
47	4,721	sp|O75390|CISY_HUMAN	Citrate synthase, mitochondrial OS=Homo sapiens OX=9606 GN=CS PE=1 SV=2	2
48	6,684	sp|P11766|ADHX_HUMAN	Alcohol dehydrogenase class-3 OS=Homo sapiens OX=9606 GN=ADH5 PE=1 SV=4	2
49	7,726	sp|P22695|QCR2_HUMAN	Cytochrome b-c1 complex subunit 2, mitochondrial OS=Homo sapiens OX=9606 GN=UQCRC2 PE=1 SV=3	2
51	2,679	sp|Q14574|DSC3_HUMAN	Desmocollin-3 OS=Homo sapiens OX=9606 GN=DSC3 PE=1 SV=3	2
58	1,639	sp|Q96C24|SYTL4_HUMAN	Synaptotagmin-like protein 4 OS=Homo sapiens OX=9606 GN=SYTL4 PE=1 SV=2	2

Supplementary Table S3. List of peptides from Creatine Kinase M (CKM) and creatine Kinase-S (CKS) isoforms identified in the three spots selected from the 2D-gels of adipose tissue from non-COVID and post-COVID patients, as well as the post-translational modifications found in some of these peptides.

	Names	Sequence	Modifications	Protein Modifications
**Non-COVID Spot 1**	**Creatine kinase M-type**	GQSIDDMIPAQK	Oxidation(M)@7	
		LGSSEVEQVQLVVDGVK		
		LSVEALNSLTGEFK		
		LSVEALNSLTGEFKGK		
		RGTGGVDTAAVGSVFDVSNADR		
		RGTGGVDTAAVGSVFDVSNADRLGSSEVEQVQLVVDGVK		
		SFLVWVNEEDHLR		
		SFLVWVNEEDHLRVISMEK	Oxidation(M)@17	

		TDLNHENLKGGDDLDPNYVLSSR		
		VLTLELYK		
		FEEILTR		
**Non-COVID Spot 2**	**Creatine kinase M-type**	GGDDLDPNYVLSSR		
		GQSIDDMIPAQK	Oxidation(M)@7	
		LGSSEVEQVQLVVDGVK		
		LSVEALNSLTGEFK		
		TDLNHENLKGGDDLDPNYVLSSR		
		SFLVWVNEEDHLRVISMEK	Oxidation(M)@17	
		SFLVWVNEEDHLR		
		VLTLELYK		
**Non-COVID Spot 3**	**Creatine kinase M-type**	GQSIDDMIPAQK	Oxidation(M)@7	
		LNYKPEEEYPDLSK		
		LSVEALNSLTGEFK		
		RGTGGVDTAAVGSVFDVSNADR		
		TDLNHENLKGGDDLDPNYVLSSR		
		VLTLELYK		
		FEEILTR		
		GGDDLDPNYVLSSR		
		ELFDPIISDR		
	**Names**	**Sequence**	**Modifications**	**Protein Modifications**
**Post-COVID Spot 1**	**Creatine kinase M-type**	AGHPFMWNQHLGYVLTCPSNLGTGLR	Carbamidomethyl(C)@17	
		ELFDPIISDR		
		EQQQLIDDHFLFDKPVSPLLLASGMAR		
		FCVGLQK	Carbamidomethyl(C)@2	
		GGDDLDPNYVLSSR		
		GQSIDDMIPAQK	Oxidation(M)@7	
		GTGGVDTAAVGSVFDVSNADRLGSSEVEQVQLVVDGVK		
		GYTLPPHCSR	Carbamidomethyl(C)@8	
		LGSSEVEQVQLVVDGVK		

		LNYKPEEEYPDLSK		
		LSVEALNSLTGEFK	Deamidated(N)@7	
		LSVEALNSLTGEFKGK		
		RGTGGVDTAAVGSVFDVSNADR		
		RGTGGVDTAAVGSVFDVSNADRLGSSEVEQVQLVVDGVK		
		SFLVWVNEEDHLR		
		SMTEKEQQQLIDDHFLFDKPVSPLLLASGMAR	Oxidation(M)@2; Oxidation(M)@30	
		TDLNHENLKGGDDLDPNYVLSSR		
		VLTLELYK		
		VLTLELYKK		
		LEKGQSIDDMIPAQK	Carbamidomethyl(K)@3; Oxidation(M)@10	
		SFLVWVNEEDHLRVISMEK		
		FEEILTR		
		DKETPSGFTVDDVIQTGVDNPGHPFIM	Oxidation(M)@27	
		TVGCVAGDEESYEVFKELFDPIISDR	Carbamidomethyl(C)@4	
		VLTCPSNLGTGLR	Carbamidomethyl(C)@4	
		AVEKLSVEALNSLTGEFK	Carbamidomethyl(K)@4	
		IEEIFK		
		KELFDPIISDR		
		AGHPFMWNQHLGY	Oxidation(M)@6	
		DVSNADRLGSSEVEQVQLVVDGVK		
		NYKPEEEYPDLSK		
		HPKFEEILTR	Carbamidomethyl(K)@3	
		GTGGVDTAAVGSVFDVSNADR	Cation:Na(D)@6	
		SIKGYTLPPHCSR	Carbamyl(K)@3; Carbamidomethyl(C)@11	
		IEEIFKK		
		FKLNYKPEEEYPDLSK	Carbamidomethyl@N-term	
		LVWVNEEDHLR		

		LMVEMEK	Oxidation(M)@2	
		FCVGLQKIEEIFK	Carbamidomethyl(C)@2; Carbamidomethyl(K)@7	
		GGVHVKLAHLSK	Carbamyl(K)@6	
		SIDDMIPAQK	Oxidation(M)@5	
		TLELYKK		
		AVEKLSVEALNSLTGEFKGK	Phospho(K)@4; Phospho(S)@6; Carbamidomethyl(K)@20	Phospho(K)@156; Phospho(S)@158
		FCVGLQKIEEIFKK	Carbamidomethyl(C)@2; Carbamidomethyl(K)@7	
**Post-COVID Spot 2**	**Creatine kinase M-type**	AGHPFMWNQHLGYVLTCPSNLGTGLR	Oxidation(M)@6; Dioxidation(W)@7; Carbamidomethyl(C)@17	
		ELFDPIISDR		
		GGDDLDPNYVLSSR		
		GQSIDDMIPAQK	Oxidation(M)@7	
		GTGGVDTAAVGSVFDVSNADR		
		GTGGVDTAAVGSVFDVSNADRLGSSEVEQVQLVVDGVK		
		GYTLPPHCSR	Carbamidomethyl(C)@8	
		LEKGQSIDDMIPAQK	Carbamidomethyl@N-term; Oxidation(M)@10	
		LGSSEVEQVQLVVDGVK		
		LNYKPEEEYPDLSK		
		LSVEALNSLTGEFK		
		LSVEALNSLTGEFKGK	Deamidated(N)@7	
		RGTGGVDTAAVGSVFDVSNADR		
		RGTGGVDTAAVGSVFDVSNADRLGSSEVEQVQLVVDGVK		
		SFLVWVNEEDHLR	Dioxidation(W)@5	
		SFLVWVNEEDHLRVISMEK		
		SMTEKEQQQLIDDHFLFDKPVSPLLLASGMAR		

		TDLNHENLKGGDDLDPNYVLSSR		
		VLTLELYK		
		VLTLELYKK		
		EQQQLIDDHFLFDKPVSPLLLASGMAR		
		FKLNYKPEEEYPDLSK	Carbamidomethyl@N-term	
		FCVGLQK	Carbamidomethyl(C)@2	
		KAGHPFMWNQHLGYVLTCPSNLGTGLR	Oxidation(M)@7;<br>Carbamidomethyl(C)@18	
		TDLNHENLK		
		HPKFEEILTR	Carbamidomethyl(K)@3	
		GYTLPPHCSRGER	Carbamidomethyl(C)@8	
		FEEILTR		
		LNYKPEEEYPDLSKHNNHMAK	Deamidated(N)@17;<br>Oxidation(M)@19	
		NYKPEEEYPDLSK		
		VLTCPSNLGTGLR	Carbamidomethyl(C)@4	
		IEEIFKK		
		TDLNHENLKGGDDLD		
		AGHPFMWNQHLGY		
		KELFDPIISDR		
		SMTEKEQQQLIDDHFLFDKPVSPLLLASGMARDWPDAR	Oxidation(M)@2;<br>Oxidation(M)@30;<br>Oxidation(P)@35	
		LVWVNEEDHLR		
		SIKGYTLPPHCSR	Carbamidomethyl(K)@3;<br>Carbamidomethyl(C)@11	
		GGVHVKLAHLSK	Formyl(K)@6	
		GGVHVKLAHLSKHPK		
		GDDLDPNYVLSSR		
		DKETPSGFTVDDVIQTGVDNPGHPFIM	Oxidation(M)@27	
		LMVEMEK	Oxidation(M)@2	

		SVEALNSLTGEFKGK		
		YKPEEEYPDLSK		
		HGGYKPTDKHKTDLNHENLK	Pro->pyro-Glu(P)@6	
		SIDDMIPAQK	Oxidation(M)@5	
		AVEKLSVEALNSLTGEFKGK	Carbamidomethyl@N-term	
		TVGCVAGDEESYEVFKELFDPIISDR	Carbamidomethyl(C)@4	
		FCVGLQKIEEIFKK	Carbamidomethyl(C)@2; Carbamidomethyl(K)@7	
		EQQQLIDDHFLFDKPVSPLLLASGMARDWPDAR	Oxidation(M)@25; Oxidation(P)@30	
		VWVNEEDHLR		
		FCVGLQKIEEIFK	Carbamidomethyl(C)@2; Carbamidomethyl(K)@7	
		AGHPFMWNQHLGYVLTCPSNL	Oxidation(M)@6; Carbamidomethyl(C)@17	
		LTLELYKK		
		TLELYKK		
		AVEKLSVEALNSLTGEFK	Carbamidomethyl@N-term; Phospho(S)@6	Phospho(S)@158
	**Creatine kinase S-type**	EVENVAITALEGLK		
		GTGGVDTAAVADVYDISNIDR		
		ITQGQFDEHYVLSSR		
		LFPPSADYPDLRK		
		LGYILTCPSNLGTGLR	Carbamidomethyl(C)@7	
		REVENVAITALEGLK		
		RGTGGVDTAAVADVYDISNIDR		
		TVGMVAGDEESYEVFADLFDPVIK	Oxidation(M)@4	
		TFLIWINEEDHTR		
		GLSLPPACTR	Carbamidomethyl(C)@8	
		LFPPSADYPDLR		
		AERREVENVAITALEGLK		

		VTPNGYTLDQCIQTGVDNPGHPFIK	Deamidated(N)@4; Carbamidomethyl(C)@11	
		GWEFMWNER	Oxidation(M)@5	
		HNNCMAECLTPAIYAK	Carbamidomethyl(C)@4; Oxidation(M)@5; Carbamidomethyl(C)@8	
**Post-COVID Spot 3**	**Creatine kinase M-type**	AGHPFMWNQHLGYVLTCPSNLGTGLR	Carbamidomethyl(C)@17	
		DKETPSGFTVDDVIQTGVDNPGHPFIM	Oxidation(M)@27	
		DVSNADRLGSSEVEQVQLVVDGVK		
		ELFDPIISDR		
		EQQQLIDDHFLFDKPVSPLLLASGMAR	Glu->pyro-Glu@N-term; Oxidation(M)@25	
		ETPSGFTVDDVIQTGVDNPGHPFIM	Oxidation(M)@25	
		FCVGLQK	Carbamidomethyl(C)@2	
		GGDDLDPNYVLSSR		
		GQSIDDMIPAQK		
		GTGGVDTAAVGSVFDVSNADR		
		GTGGVDTAAVGSVFDVSNADRLGSSEVEQVQLVVDGVK		
		GYTLPPHCSR	Carbamidomethyl(C)@8	
		LGSSEVEQVQLVVDGVK		
		LGSSEVEQVQLVVDGVKL	Oxidation(K)@17; Amidated@C-term	Oxidation(K)@358
		LNYKPEEEYPDLSK	Deamidated(N)@2	
		LNYKPEEEYPDLSKHNNHMAK	Deamidated(N)@16; Oxidation(M)@19	
		LSVEALNSLTGEFK	Deamidated(N)@7	
		LSVEALNSLTGEFKGK		
		QHLGYVLTCPSNLGTGLR	Carbamidomethyl(C)@9	
		RGTGGVDTAAVGSVFDVSNADR		
		RGTGGVDTAAVGSVFDVSNADRLGSSEVEQVQLVVDGVK		
		SFLVWVNEEDHLR		
		SMTEKEQQQLIDDHFLFDKPVSPLLLASGMAR	Oxidation(M)@30	

		TDLNHENLKGGDDLDPNYVLSSR		
		VLTLELYKK		
		VLTLELYK		
		SVEALNSLTGEFKGK		
		AVEKLSVEALNSLTGEFK	Carbamidomethyl(K)@4	
		FEEILTR		
		LRDKETPSGFTVDDVIQTGVDNPGHPFIM		
		MWNQHLGYVLTCPSNLGTGLR	Oxidation(M)@1; Carbamidomethyl(C)@12	
		FCVGLQKIEEIFKK	Carbamidomethyl(C)@2; Carbamidomethyl(K)@7	
		LMVEMEK	Oxidation(M)@2; Oxidation(M)@5	
		TVGCVAGDEESYEVFKELFDPIISDR	Carbamidomethyl(C)@4	
		AGHPFMWNQHLGY	Oxidation(M)@6	
		TVGCVAGDEESYEVFK	Carbamidomethyl(C)@4	
		FKLNYKPEEEYPDLSK	Carbamidomethyl@N-term	
		KELFDPIISDR		
		LEKGQSIDDMIPAQK	Carbamidomethyl(K)@3; Oxidation(M)@10	
		SIKGYTLPPHCSR	Carbamidomethyl(K)@3; Carbamidomethyl(C)@11	
		GGVHVKLAHLSK	Carbamidomethyl(K)@6	
		DKETPSGFTVDDVIQTGVDNPGHPF		
		HPKFEEILTR	Carbamidomethyl(K)@3	
		AVGSVFDVSNADR		
		LSVEALNSLTGEFKGKYYPLK		
		LGYVLTCPSNLGTGLR	Carbamidomethyl(C)@7	
		NYKPEEEYPDLSK		
		HGGYKPTDKHKTDLNHENLK	Methyl(H)@10	Methyl(H)@106
		YKPEEEYPDLSK		

		VLTCPSNLGTGLR	Carbamidomethyl(C)@4	
		SMTEKEQQQLIDDHFLFDKPVSPLLLASGMARDWPDAR	Oxidation(M)@2;<br>Oxidation(M)@30;<br>Oxidation(P)@35	Oxidation(P)@212
		GDDLDPNYVLSSR		
		SIDDMIPAQK	Oxidation(M)@5	
		TLELYKK		
		EQQQLIDDHFLFDKPVSPLLLASGMARDWPDAR	Oxidation(M)@25;<br>Oxidation(W)@29	
		ENLKGGDDLDPNYVLSSR		
		FCVGLQKIEEIFK	Carbamidomethyl(C)@2;<br>Carbamidomethyl(K)@7	
		VAGDEESYEVFK		
		DLDPNYVLSSR		
		TDLNHENLKGGDDLD		
		VAGDEESYEVFKELFDPIISDR		
		LTLELYKK		
		VWVNEEDHLR		
	**Creatine kinase S-type**	EVENVAITALEGLK		
		ITQGQFDEHYVLSSR		
		LFPPSADYPDLRK		
		LGYILTCPSNLGTGLR	Carbamidomethyl(C)@7	
		LIDDHFLFDKPVSPLLTCAGMAR	Carbamidomethyl(C)@18;<br>Oxidation(M)@21	
		TFLIWINEEDHTR		
		TVGMVAGDEESYEVFADLFDPVIK	Oxidation(M)@4	
		VTPNGYTLDQCIQTGVDNPGHPFIK	Carbamidomethyl(C)@11	
		GTGGVDTAAVADVYDISNIDR		
		HNNCMAECLTPAIYAK	Carbamidomethyl(C)@4;<br>Oxidation(M)@5;<br>Carbamidomethyl(C)@8	

		LFPPSADYPDLR		
		GLSLPPACTR	Carbamidomethyl(C)@8	
		GWEFMWNERLGYILTCPSNLGTGLR	Oxidation(M)@5; Trp->Kynurenin(W)@6; Deamidated(N)@7; Carbamidomethyl(C)@16	
